# *Cryptococcus neoformans* can form titan-like cells *in vitro* in response to multiple signals

**DOI:** 10.1371/journal.ppat.1007007

**Published:** 2018-05-18

**Authors:** Nuria Trevijano-Contador, Haroldo Cesar de Oliveira, Rocío García-Rodas, Suélen Andreia Rossi, Irene Llorente, Ángel Zaballos, Guilhem Janbon, Joaquín Ariño, Óscar Zaragoza

**Affiliations:** 1 Mycology Reference Laboratory, National Centre for Microbiology, Instituto de Salud Carlos III, Majadahonda, Madrid, Spain; 2 Universidade Estadual Paulista (UNESP), Faculdade de Ciências Farmacêuticas, Câmpus Araraquara, Departamento de Análises Clínicas, Laboratório de Micologia Clínica, Araraquara, São Paulo, Brazil; 3 Genomics Unit, Core Scientific Services, Instituto de Salud Carlos III, Majadahonda, Madrid, Spain; 4 Institut Pasteur, Unité Biologie des ARN des Pathogènes Fongiques, Département de Mycologie, Paris, France; 5 Institut de Biotecnologia i Biomedicina and Departament de Bioquímica i Biologia Molecular, Universitat Autònoma de Barcelona, Cerdanyola del Vallès, Spain; Carnegie Mellon University, UNITED STATES

## Abstract

*Cryptococcus neoformans* is an encapsulated pathogenic yeast that can change the size of the cells during infection. In particular, this process can occur by enlarging the size of the capsule without modifying the size of the cell body, or by increasing the diameter of the cell body, which is normally accompanied by an increase of the capsule too. This last process leads to the formation of cells of an abnormal enlarged size denominated titan cells. Previous works characterized titan cell formation during pulmonary infection but research on this topic has been hampered due to the difficulty to obtain them *in vitro*. In this work, we describe *in vitro* conditions (low nutrient, serum supplemented medium at neutral pH) that promote the transition from regular to titan-like cells. Moreover, addition of azide and static incubation of the cultures in a CO_2_ enriched atmosphere favored cellular enlargement. This transition occurred at low cell densities, suggesting that the process was regulated by quorum sensing molecules and it was independent of the cryptococcal serotype/species. Transition to titan-like cell was impaired by pharmacological inhibition of PKC signaling pathway. Analysis of the gene expression profile during the transition to titan-like cells showed overexpression of enzymes involved in carbohydrate metabolism, as well as proteins from the coatomer complex, and related to iron metabolism. Indeed, we observed that iron limitation also induced the formation of titan cells. Our gene expression analysis also revealed other elements involved in titan cell formation, such as calnexin, whose absence resulted in appearance of abnormal large cells even in regular rich media. In summary, our work provides a new alternative method to investigate titan cell formation devoid the bioethical problems that involve animal experimentation.

## Author summary

*Cryptococcus neoformans* is a fungal pathogen that has a significant incidence in HIV+ patients in particular, in Sub-saharan Africa, Asia and South America. This yeast poses an excellent model to investigate fungal virulence because it develops many strategies to adapt to the host and evade the immune response. One of the adaptation mechanisms involves the formation of Titan Cells, which are yeast of an abnormal large size. However, research on these cells has been limited to *in vivo* studies (mainly in mice) because they were not reproducibly found *in vitro*. In this work, we describe several conditions that induce the appearance of cells that mimic titan cells, and that we denominated as titan-like cells. The main factor that induced titan-like cells was the addition of serum to nutrient limited media. This has allowed to easily performing new approaches to characterize several signaling pathways involved in their development. We found that the formation of these cells is regulated by quorum sensing molecules, and that pathways such as cAMP and PKC regulate the process of cellular enlargement. We have also performed transcriptomic analysis, which led to the identification of new genes that could be involved in the process. This work will open different research lines that will contribute to the elucidation of the role of these cells during infection and on the development of cryptococcal disease.

## Introduction

*Cryptococcus neoformans* is a basidiomycetes yeast widely distributed in the environment that can behave as a pathogen in susceptible patients [1, 2]. *Cryptococcus neoformans* can survive in the lung, but in immunosuppressed patients it can also spread to the central nervous system and cause meningoencephalitis [2]. Cryptococcal infections are major causes of death in HIV patients. Although the incidence has significantly decreased in developed countries due to the introduction of antiretroviral therapy (ART), associated mortality remains high [3, 4]. Moreover, infections by this yeast still present a high incidence in developing areas, such as the sub-Saharan Africa and Southeast Asia [5, 6].

One of the most characteristic features of *Cryptococcus* is its ability to adapt to the lung environment and to evade the host immune response. Several factors contributing to cryptococcal adaptation to the lung have been described. The most important is the presence of a polysaccharide capsule [7–9], which is antiphagocytic and protects the yeasts from stress conditions [2, 10]. The size of the capsule is not constant, and it increases during the first hours of interaction with the host [11], which indicates that this process is a response that contributes to immune evasion. Furthermore, the capsular polysaccharide is also secreted to the extracellular media where it induces immunological paralysis through multiple mechanisms [9, 12–15]. *Cryptococcus neoformans* is also a facultative intracellular pathogen in phagocytic cells [16–19], which is another important factor that contributes to fungal survival in the host.

*Cryptococcus* has also developed other adaptation mechanisms that contribute to the evasion of the immune response. One of them involves the formation of titan cells, which have an abnormal large size. The average diameter of *Cryptococcus* cells grown *in vitro* ranges between 4–7 microns. In contrast, the fungal population in the lungs is very heterogeneous, and cells of even 100 microns have been described [11, 20–22]. Titan cells have been arbitrarily defined as those with a cell body diameter above 15 microns or with a total size (capsule included) over 30 microns [23]. Because of their size, titan cells cannot be phagocytosed, and they can persist in the host for long periods [24, 25]. Titan cells also contribute to virulence through other mechanisms. For example, they can divide and produce a progeny of regular size that has increased resistance to stress factors [26] as well as the ability to inhibit the phagocytosis of cells of regular size [25].

Some signals and pathways involved in titan cell formation have been characterized, and it is known that the cAMP pathway is required for this phenomenon [20, 21, 27]. Titan cell formation has been associated *in vivo* with anti-inflammatory Th2 type immune responses [23], but the host's factors that trigger this morphological transition remain unknown.

The investigation and characterization of titan cells has been limited by the lack of media allowing the transition *in vitro*, so most of the data about these cells has been obtained using animal models. Although this approach has been shown to be useful for some purposes, it does not allow obtaining a large population of titan cells. In addition, the use of mice for these purposes presents associated significant bioethical issues. In this work, we have defined *in vitro* conditions that induce cell enlargement in *C*. *neoformans*, which lead to the appearance of cells similar to those found *in vivo*, here denominated *titan-like cells*. We found that incubation of this fungus in low nutrient media supplemented with serum in a CO_2_ enriched atmosphere induced cryptococcal cell size increase. Moreover, other factors, such as oxygen limitation, or low cell density enhanced the cell growth. We have used this medium as a first step in the characterization of this transition. Our findings open future research lines that will help to define the molecular mechanisms that trigger titan cell formation and their role during infection.

## Results

### *Cryptococcus neoformans* can significantly enlarge its cell size *in vitro*

**Fig 1:**
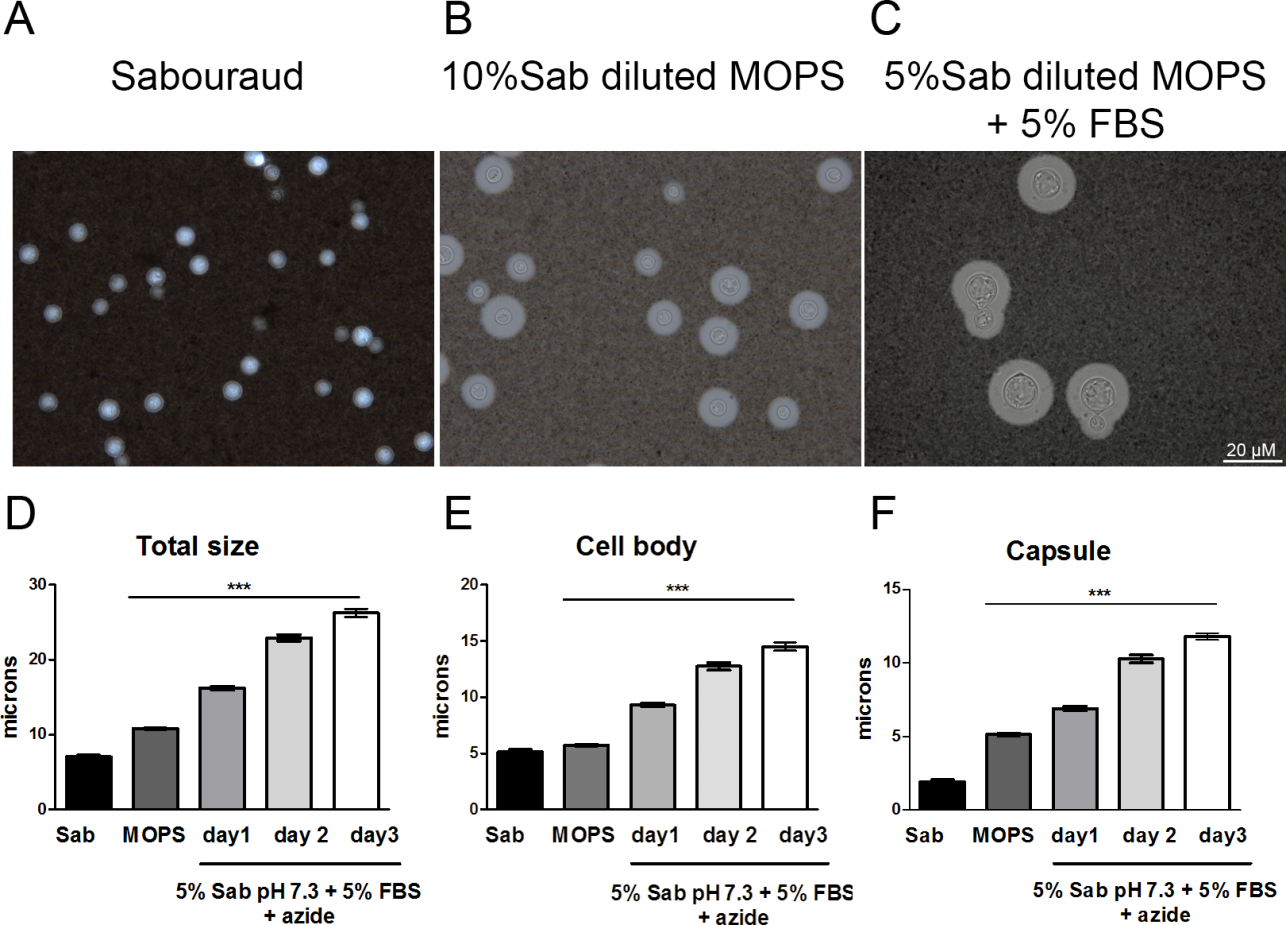
Cellular and capsular size of *C*. *neoformans* in different media. Cells from strain H99 were inoculated into Sabouraud (A), capsule inducing medium (10% Sabouraud with MOPS at pH 7.3) (B) and 5% Sabouraud with MOPS + 5% FBS + sodium azide (C). After incubation at 37°C with shaking, total size and capsule size was visualized by suspending the cells in India ink. Total size (D), cell body size (E) and capsule size (F) distribution after incubation as described above. The asterisks indicate significant differences compared to Sabouraud control (p<0.05). Sab, Sabouraud medium.

In the last years, we have characterized the phenomenon of capsule growth *in vitro* using a medium that contains 10% Sabouraud buffered at pH 7.3 with 50 mM MOPS. In a set of experiments, we observed that addition of serum and of the respiration inhibitor sodium azide to this medium produced not only growth of the capsule but also of the cell body (Fig 1A–1C). These cells resembled the titan cells observed *in vivo*, although they did not reach the same size. Despite this difference, we argued that this phenomenon might reflect the first steps of titan cell formation, so we decided to characterize the factors that induced *in vitro* cell growth. First, the morphology of *C*. *neoformans* was analyzed in regular growth conditions (liquid Sabouraud), in capsule inducing medium (10% Sabouraud buffered with 50 mM MOPS) and in 5% Sabouraud buffered with MOPS and 5% FBS + azide (15 μM). Titan cells have been defined as those with total diameter of 30 μm or those with a cell body diameter larger than 15 μm [22]. As shown in Fig 1D, we observed a significant increase of both the cell size and the capsule in this last medium, almost reaching the threshold for titan cells definition after three days of culture. For this reason, we decided to name the medium as TCM (Titan Cell Medium).

### Characterization of factors that induce cellular growth *in vitro*

**Fig 2:**
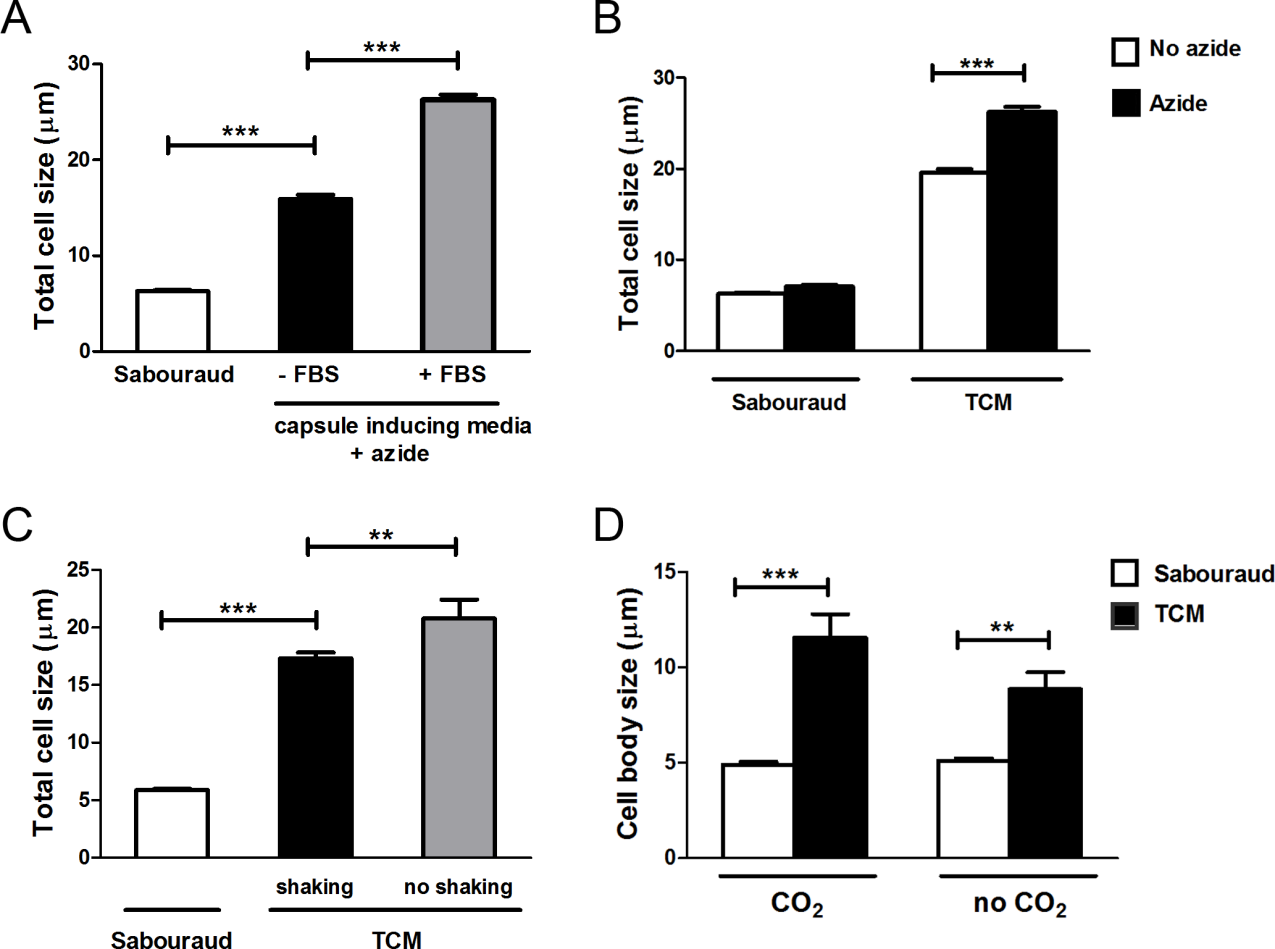
Effect of different factors on the cellular growth of *C*. *neoformans*. Cells from H99 strain were incubated on Sabouraud overnight and transferred to different media to evaluate the effect of several factors on the titan-like cell formation. (A) Cells were incubated in Sabouraud or capsule inducing medium (10% Sab, pH 7.3) supplemented with 15 μM sodium azide in the presence or absence of 5% serum (FBS) and cultures were incubated at 37°C overnight. Pictures after suspension of the cells in India Ink were taken, and the total cell size was measured and plotted (B) Effect of sodium azide (black bars) on cell size in Sabouraud or TCM medium. As a control, the same media were inoculated without sodium azide (white bars). Cells were incubated at 37°C overnight. Pictures after suspension of the cells in India Ink were taken, and the total cell size was measured and plotted (C) Effect of shaking effect on titan-like cell formation. The yeasts were incubated at 37°C for 24 h in flasks with Sabouraud or TCM medium in both conditions (black bars, shaking) and (gray bars, no shaking). Pictures after suspension of the cells in India Ink were taken, and the total cell size was measured and plotted (D) Effect of CO_2_ on cell growth. The cells were incubated in Sabouraud (white bars) and TCM (black bars) with and without 5% of CO_2_. In both cases, the cells were grown at 37°C without shaking for 24 h in a 96-microdilution plate. Then, the plate was directly observed in the microscope, and the cell body size of was measured and plotted.

Serum was essential for cellular enlargement formation because in its absence the increase in cell size was significantly lower (p<0.05, Fig 2A). We characterized in detail the factors and conditions that favor cryptococcal cell size increase *in vitro*. In our initial experiments, the medium in which we first observed cells of enlarged size contained subinhibitory concentrations of the mitochondrial inhibitor sodium azide to prevent contamination. As shown in Fig 2B, the process of cell enlargement was enhanced in the presence of sodium azide. This compound is an inhibitor of complex IV of the respiratory chain, so we argued that other factors that alter respiration could induce increases in cell size. For this reason, we investigated whether cryptococcal cell growth was influenced by oxygen limitation. To assess that, we compared the yeast morphology under shaking or static conditions. As shown in Fig 2C, static incubation of the cultures resulted in a greater proportion of cells with enlarged size.

Cryptococcal cells sense and respond to environmental levels of CO_2_ and it is known that this molecule induces capsule growth. For this reason, we investigated if incubation of the cultures in a CO_2_-enriched environment altered cryptococcal cell size. We found that yeast size was significantly larger when the plates were placed in a 5% CO_2_ atmosphere in comparison to growth without CO_2_ (Fig 2D).

To visualize the phenomenon of cell growth, we carried out *in vivo* imaging by placing the cells in a 96-wells plate in TCM in 5% CO_2_ at 37°C under a microscope overnight and obtained videos of the cellular enlargement. As shown in S1 Video, cells actively grew and replicated in Sabouraud medium. However, in TCM, after 5–8 h of incubation the cells started to enlarge during 8–10 h (S2 Video). After this time, the cells stopped enlarging and continued budding. We also observed that cellular enlargement was associated with some intracellular phenotypic changes. For instance, a significant proportion of the cells displayed an intracellular compartment that started to divide by fission, but then fused again to render a large vesicle (S3 Video).

**Fig 3:**
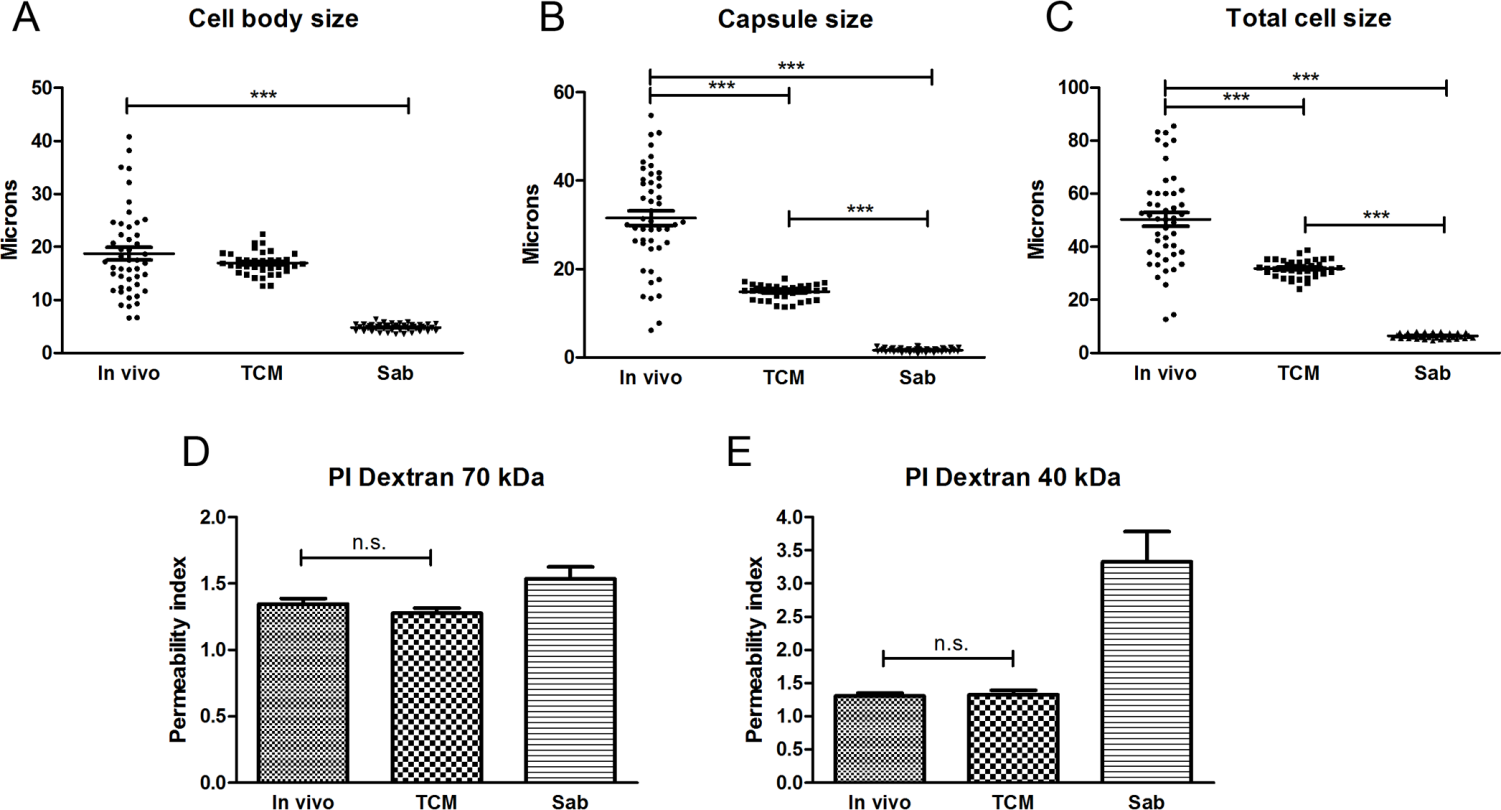
Comparison of titan cells obtained in vivo with enlarged cells obtained *in vitro*. Measurements of cell body (A), capsule (B) and total cell sizes (C) of cells isolated from infected mice (*in vivo*) and cultured in TCM and Saboraud *in vitro*. D and E show the permeability index of 70 and 40 kDa fluorescent labelled dextrans respectively in the same type of samples (see M&M).

To evaluate to which extent the cells obtained *in vitro* resembled titan cells found in animal models, we infected mice and isolated cryptococcal cells after 14 days of infection (see Material & Methods). We first determined the total size, capsule size, and cell body size of titan cells. As shown in Fig 3, the cells obtained *in vitro* in TCM did not reach the size found in the cells *in vivo*. The main difference between both types of cells was at the capsule, being it size significantly larger in titan cells isolated from mice. For this reason, we decided to denominate the cells obtained *in vitro* as “titan-like” cells.

Titan cells also present differences in capsular features, such as the density, compared to cells obtained *in vitro* [21]. To investigate if the density of titan-like cells was similar to that observed *in vivo*, we measured the permeability index using fluorescently labeled dextrans of different molecular weights (70 and 40 kDa). As shown in Fig 3D and 3E, penetration of the dextrans in the capsule of titan-like cells was reduced compared to cells of regular size and similar to that of titan cells isolated from the lung of infected mice. This result indicated that titan-like cells had a capsule of similar density of that of titan cells generated *in vivo*.

**Fig 4:**
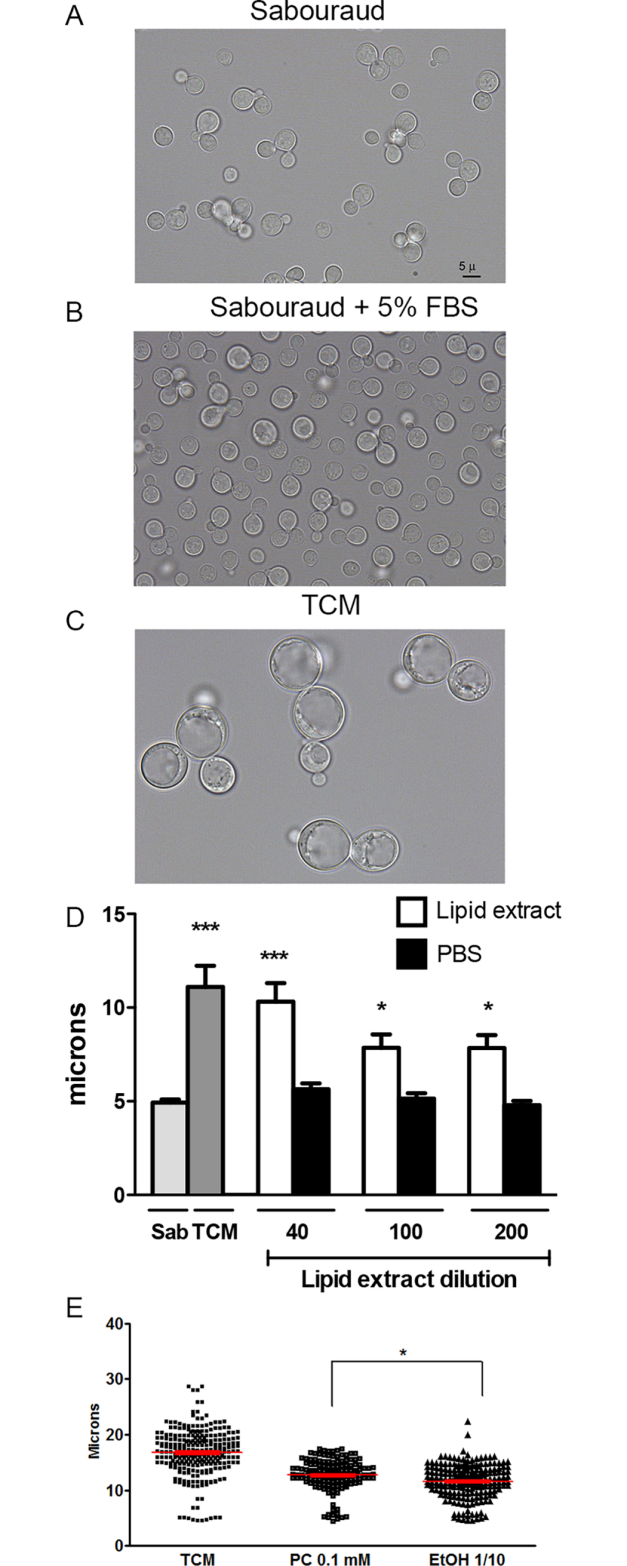
Effect of serum on cryptococcal cell growth. H99 cell suspensions were inoculated in Sabouraud (A), Sabouraud supplemented with 5% serum (B) or TCM (C). The cells were incubated overnight at 37°C in 5% CO_2_. Then, the cells were suspended in India Ink and pictures of the cell body size were taken. The scale in A applies to all the pictures. (D) Effect of purified serum lipids on cell growth. The cells (initial density 10^4^ cells/mL) were grown in TCM medium in which the serum was replaced by different dilutions (40, 100 and 200) of the lipid extract. As controls, the same media was supplemented with PBS. Cultures in TCM are also included for reference. Cell body size was determined after 24 hours at 37° C with CO_2_ and shaking. The asterisks indicate significant differences compared to Sabouraud control. (E) Effect of phosphatydilcholine on titan-like cell formation. Cells were incubated as in (A), but serum was replaced by 0.1 mM of phosphatidylcholine (PC). A parallel control with the same concentration of ethanol was carried out in parallel. The graph shows the cell body size.

Although serum was required to induce cellular enlargement *in vitro*, it was not sufficient for this process. Serum did not induce cellular growth in rich media (Fig 4A and 4B), in contrast to the situation in the diluted medium nutrients (Fig 4C), indicating that nutrient limitation was important for cell size enlargement.

### Effects of phospholipids from fetal calf serum in titan-like cell formation

Phospholipids, in particular phosphatidylcholine, can trigger the appearance of titan cells *in vitro* [28]. For this reason, we performed a lipid extraction of fetal calf serum, present in TCM and we incubated the cells with different amounts of these lipids (1/40, 1/100 and 1/200 dilution of the original lipid solution). As shown in Fig 4D, the lipids present in the serum induced titan-like cell formation.

Phosphatidylcholine (PC) is one of the major phospholipids contained in serum and mammalian membranes. For this reason, we investigated if this molecule had any effect on titan-like cell formation. We performed experiments in which serum was replaced by different concentrations of PC, and compared its effect with control samples containing the same concentration of solvent (ethanol). As shown in Fig 4E, PC induced an increase in cryptococcal cell size. However, this increase was lower compared to the one observed with serum, indicating that although PC seemed to induce titan-like cells, there are other serum components responsible for the cell increase.

### Cell density influences titan-like cell formation

**Fig 5:**
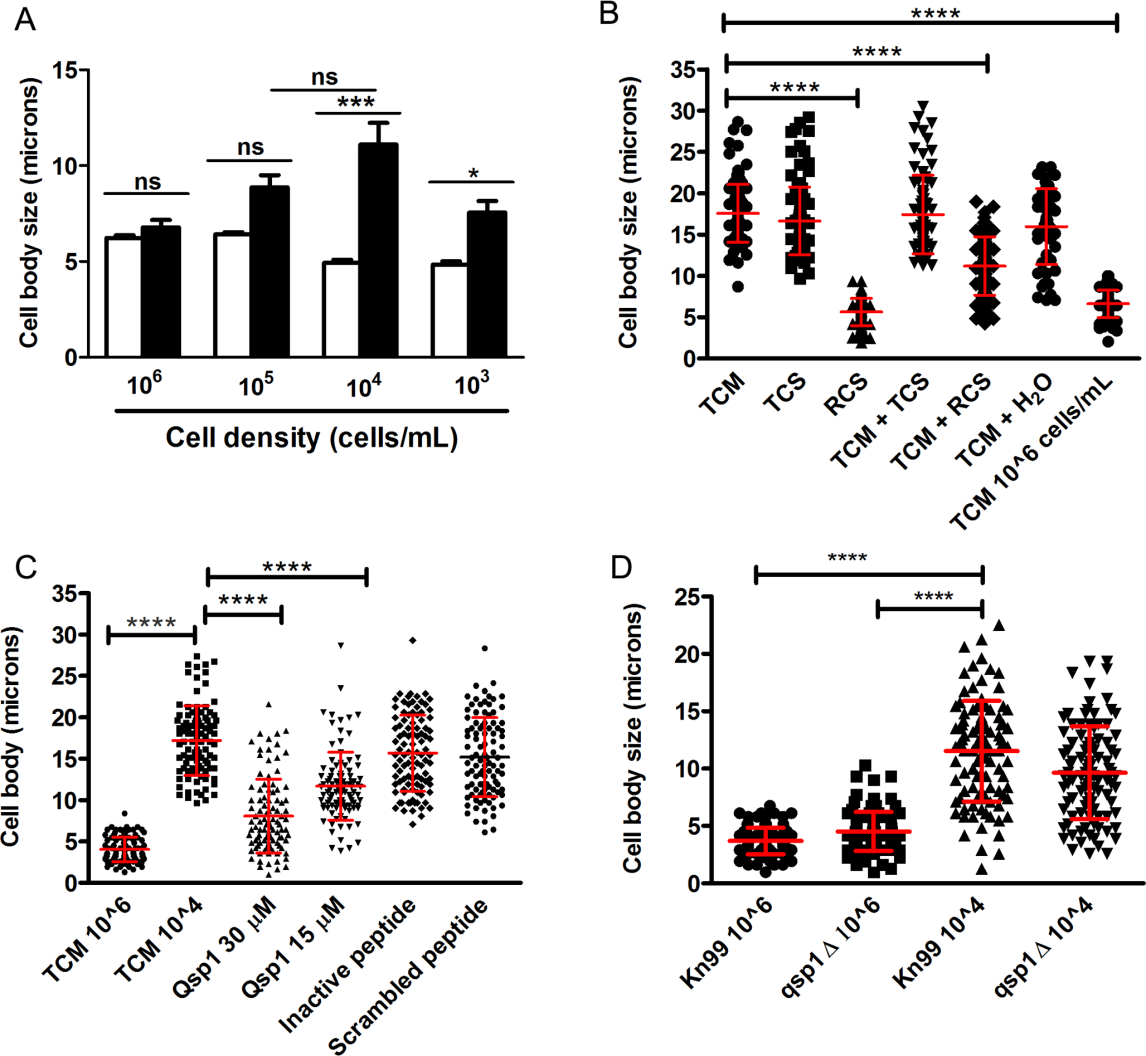
Influence of cell density on titan-like cell formation in *C*. *neoformans*. (A). Cells from H99 strain inoculated in Sabouraud (white bars) or TCM (black bars) at different concentrations (10^3^, 10^4^, 10^5^ and 10^6^ cells/mL) in 96-well plates. Cell body size was measured after incubation overnight at 37°C with CO_2._ The asterisks indicate significant differences. B) Effect of conditioned media on cell body size. Cells from H99 strain were incubated in different conditioned TCM medium denominated (see material and methods) as TCS (supernatant of titan-like cells TCM cultures), RCS (supernatant of regular size TCM cultures), TCM + TCS (a 1:1 mixture of fresh TCM with TCS), TCM + RCS (a 1:1 mixture of fresh TCM with RCS). As controls we used a diluted TCM in H_2_O (1:1) and fresh TCM inoculated with 10^4^ cells/mL (TCM) and with 10^6^ cells/mL. (C) Cells from H99 strain were grown in TCM supplemented with 30 uM and 15 uM of the quorum sensing peptide Qsp1 (NFGAPGGAYPW). As controls, TCM supplemented with 30 uM of an inactive Qsp1 (NFGAPGAAYPW), with 30 uM of a scrambled Qsp1 peptide (AWAGYFPGPNG). TCM without any supplementation was used as control. Cell body size was measured after overnight incubation at 37°C with 5% CO_2_. D) Titan-like cell formation of *qsp1* mutant. Cells from the KN99 (wild type) and *qsp1* mutant were inoculated in TCM at 10^4^ and 10^6^ cells/mL, and cell body size was determined after 18 h of incubation at 37°C in the presence of 5% CO_2_.

We found that titan-like cell formation depended on the cell density of the cultures. We inoculated 96-wells plates with different concentrations of cells from H99 strain (10^6^, 10^5^, 10^4^ and 10^3^ cells/mL) in TCM and Sabouraud as a control of growth. After overnight incubation at 37°C with CO_2_, titan-like cells were observed in the wells inoculated with 10^3^, 10^4^ and 10^5^ cells/mL but were almost absent in the wells that were inoculated with the higher cell density (10^6^ cells/mL, Fig 5A). We found that titan-like cells were observed more frequently when the cultures were inoculated with cellular concentrations around 10^4^ cells/mL.

### Effect of QS molecules on titan-like cell formation

The fact that the formation of titan-like cells depends on cell density suggests that this process could be regulated by quorum sensing (QS). QS is a cell-cell communication mechanism mediated by molecules that are released directly into the medium by microorganisms. These molecules are released as a function of growth and replication rate [29, 30]. In this way, we evaluated the influence on titan-like cell formation of cell-free media obtained from titan-like and regular *C*. *neoformans* cultures. We inoculated TCM with the H99 strain at 10^6^ cells/mL and 10^4^ cells/mL and incubated the cultures for 18 h at 37°C in 5% CO_2_ to obtain cells of regular size and titan-like cells, respectively. We then collected the supernatants (named RCS and TCS, respectively). These conditioned media were added to wells that contained fresh TCM inoculated at 10^4^ cells/mL. We found that the conditioned medium RCS significantly inhibited the formation of titan-like cells (Fig 5B) even when added to fresh TCM (TCM + RCS) (p<0.001). In contrast, the supernatant from titan-like cells cultures (TCS) did not block the formation of the titan-like cells, demonstrating a negative effect of the supernatant obtained from cells of regular sizes on titan-like cell formation (Fig 5B). The effect of the TCS conditioned medium was not explained by the dilution of the nutrients of the fresh TCM, since titan-like cells were still formed in TCM diluted with distilled water (Fig 5B).

In *C*. *neoformans*, the main QS molecule described is a short peptide (11-mer) called Qsp1 that is required for fungal virulence, replication, cell wall synthesis and protease activities [31, 32]. To investigate the influence of Qsp1 in the formation of titan-like cells, different concentrations of the peptide were added to the TCM medium and the formation of titan-like cells was evaluated. We observed that Qsp1 significantly inhibited formation of titan-like cells in a dose-dependent manner (Fig 5C). As control, we used both inactive and scrambled versions of Qsp1, and observed that none of them had any effect on titan-like cell development (Fig 5C).

It could be argued that the production of Qsp1 in TCM cultures inoculated at high cell densities was responsible for the inhibition of titan-like cell formation. To test this idea, we used a *qsp1* mutant that does not produce Qsp1 [32]. Our results showed that the mutant produced titan-like cells in a similar way as the wild type strain KN99 (Fig 5D) [32], even at high cell densities (10^6^ cells/mL). This result indicates that absence of titan-like cells in TCM cultures inoculated at high densities was not only due to Qsp1, and that most probably, other QS molecules secreted by *C*. *neoformans* might influence cellular enlargement.

### Nuclear staining

**Fig 6:**
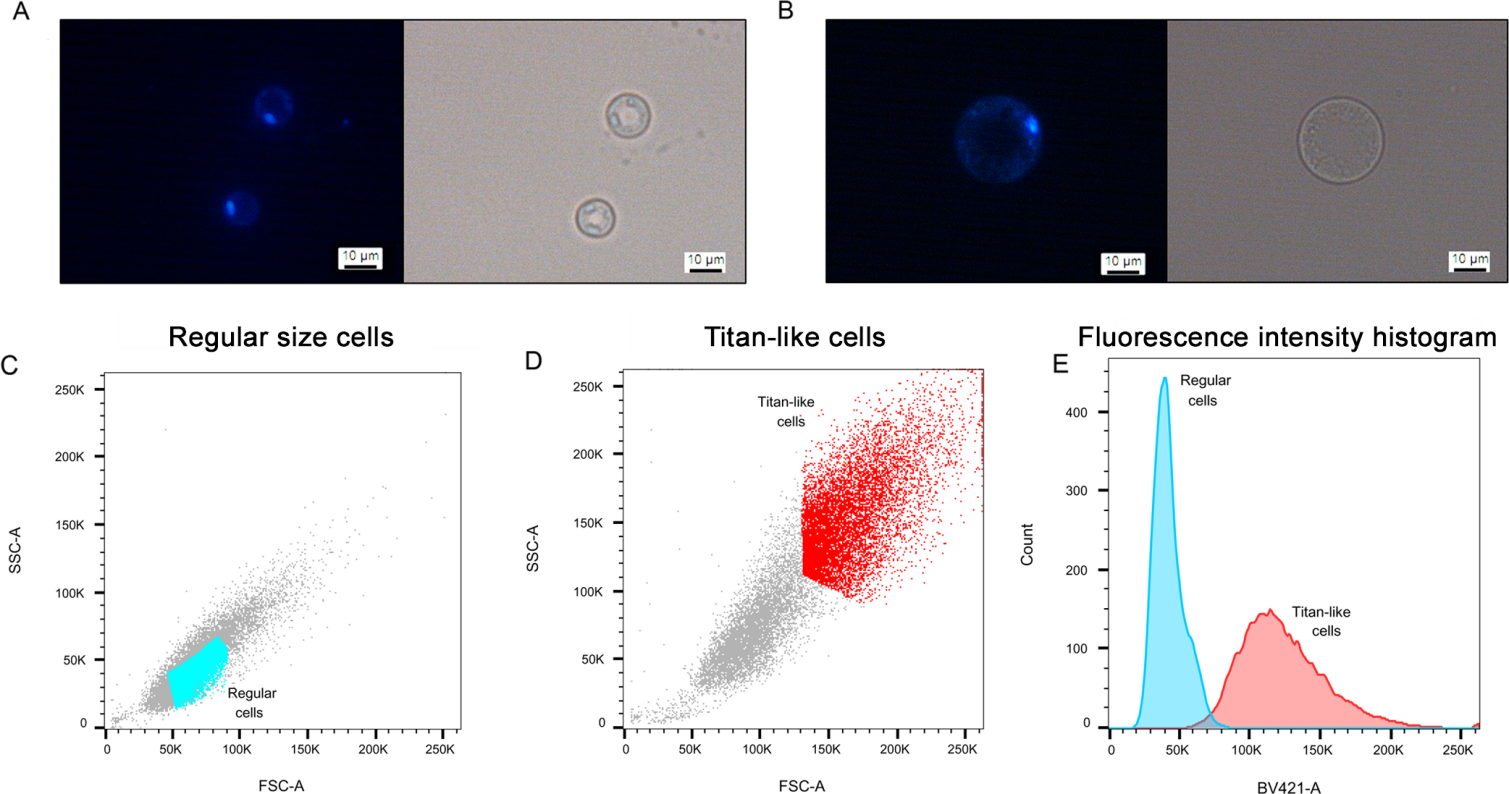
Nuclear staining of titan-like cells. Cells of regular size and titan-like cells were obtained by inoculating H99 strain in TCM at 10^6^ and 10^4^ cells/mL respectively. After incubating overnight at 37°C with 5% CO_2_, the cells were fixed and stained with DAPI as described in material and methods. A and B show the microscopic appearance of the nucleus of cells of regular size (A), and titan-like cells (B). C-E, Analysis of nuclear staining by flow cytometry. C) FFS/SSC scatter plot of cells of regular size, D) same graph of titan-like cells. In C and E, we defined a gate to clearly separate cells of regular size (cells in blue) and titan-like cells (cells in light red). The fluorescence intensity (histograms) of the cells from these two gates is represented in panel E. Blue histogram, fluorescence from the cells shown in the gate in panel C, and light red histogram, fluorescence from the cells shown in the gate in panel D.

Titan cells formed in the lungs are polyploid and single-nucleated. So we investigated the morphology of the nucleus and the DNA content after staining with DAPI. As shown in Fig 6A and 6B, titan-like cells contained one nucleus. This result was confirmed using a strain that expresses a fluorescent nucleolar protein (NOP1-mCherry, [33], S1 Fig). We also quantified the fluorescence intensity of the DAPI staining by flow cytometry. As shown in Fig 6C–6E, titan-like cells emitted more fluorescence than cells of regular size. The fluorescence intensity was more heterogeneous in titan-like cells, ranging from 2 to 5-fold increase compared to cells of normal size (Fig 6E).

### Inhibition of PKC signaling pathways affected titan-like cell formation

**Fig 7:**
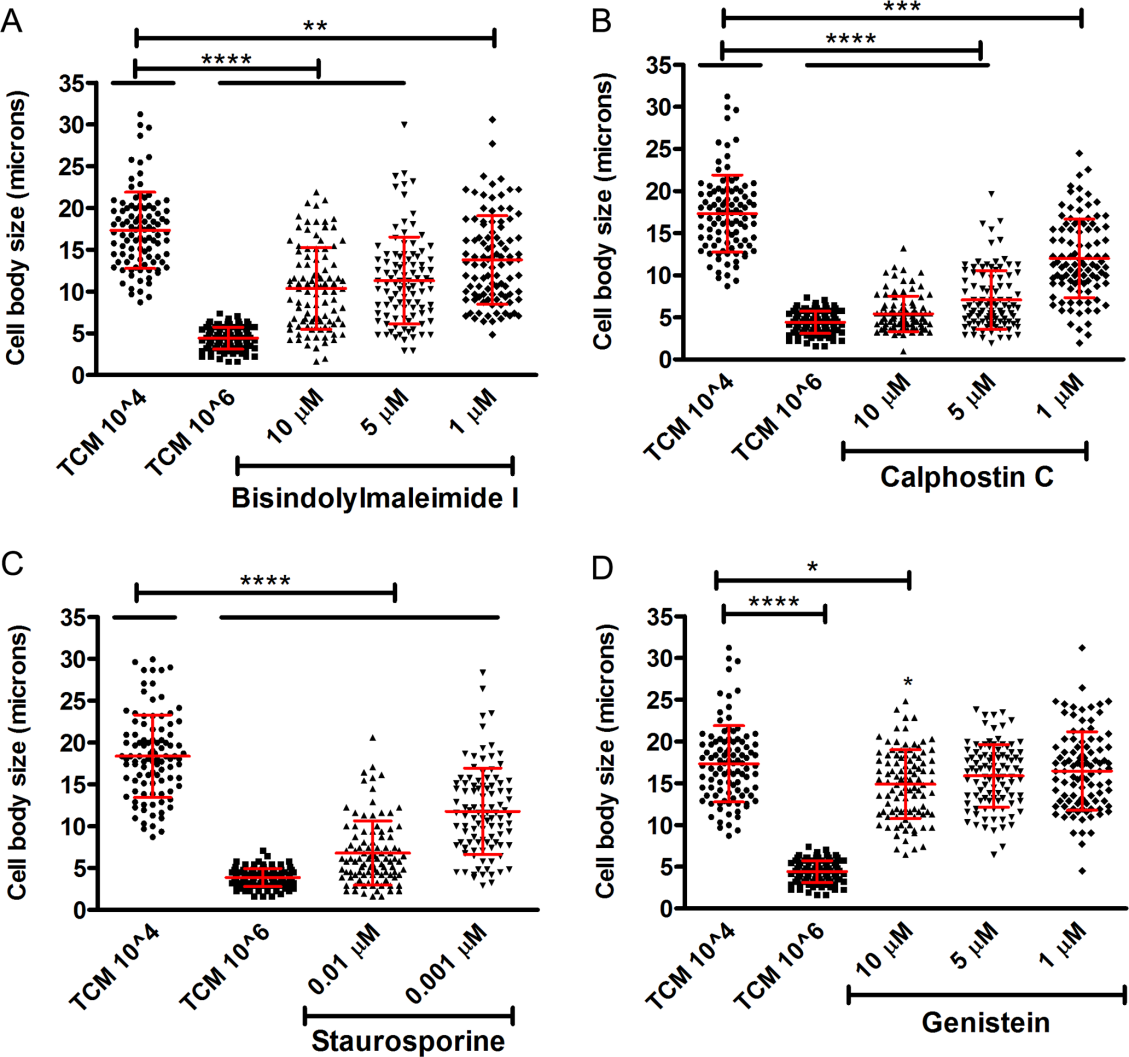
Influence of PKC pathway on titan-like cell formation. The influence of the PKC pathway was evaluated by its inhibition with three different agents: Bisindolylmaleimide I (A), Calphostin C (B), Staurosporine (C). Genestein, a tyrosine kinase inhibitor, was used as control (D). The experiments in A, B and D were performed the same days, so they share the same control. However, for clarity, the graphs corresponding to each inhibitor have been separated. The experiments were repeated on three days, and the data from the three experiments is plotted. Cells of the H99 strain were incubated with these different agents overnight in TCM at 37°C with 5% CO_2_ without shaking and cell body size was measured. The asterisks indicate statistical significant differences.

PKC is a family of protein kinases, activated by Ca^2+^, diacylglycerol (DAG) and phospholipids, and involved in different virulence-related aspects in *C*. *neoformans* such as melanin production [34], temperature tolerance, cell integrity [35] and fluconazole tolerance [36]. We argued that serum phospholipids could activate the PKC signaling pathway, so we tested the effect of three PKC inhibitors, calphostin C, staurosporine and bisindolylmaleimide I on titan-like cell formation. We observed that all PKC inhibitors (in particular, staurosporine and Calphostine C), impaired the formation of titan-like cells in a dose-dependent manner (Fig 7A–7C). We also included the tyrosine kinase inhibitor genistein and found that this compound had no visible effect on cellular enlargement (Fig 7D).

### Titan-like cell formation did not correlate with the serotype or mating type of the strain

**Fig 8:**
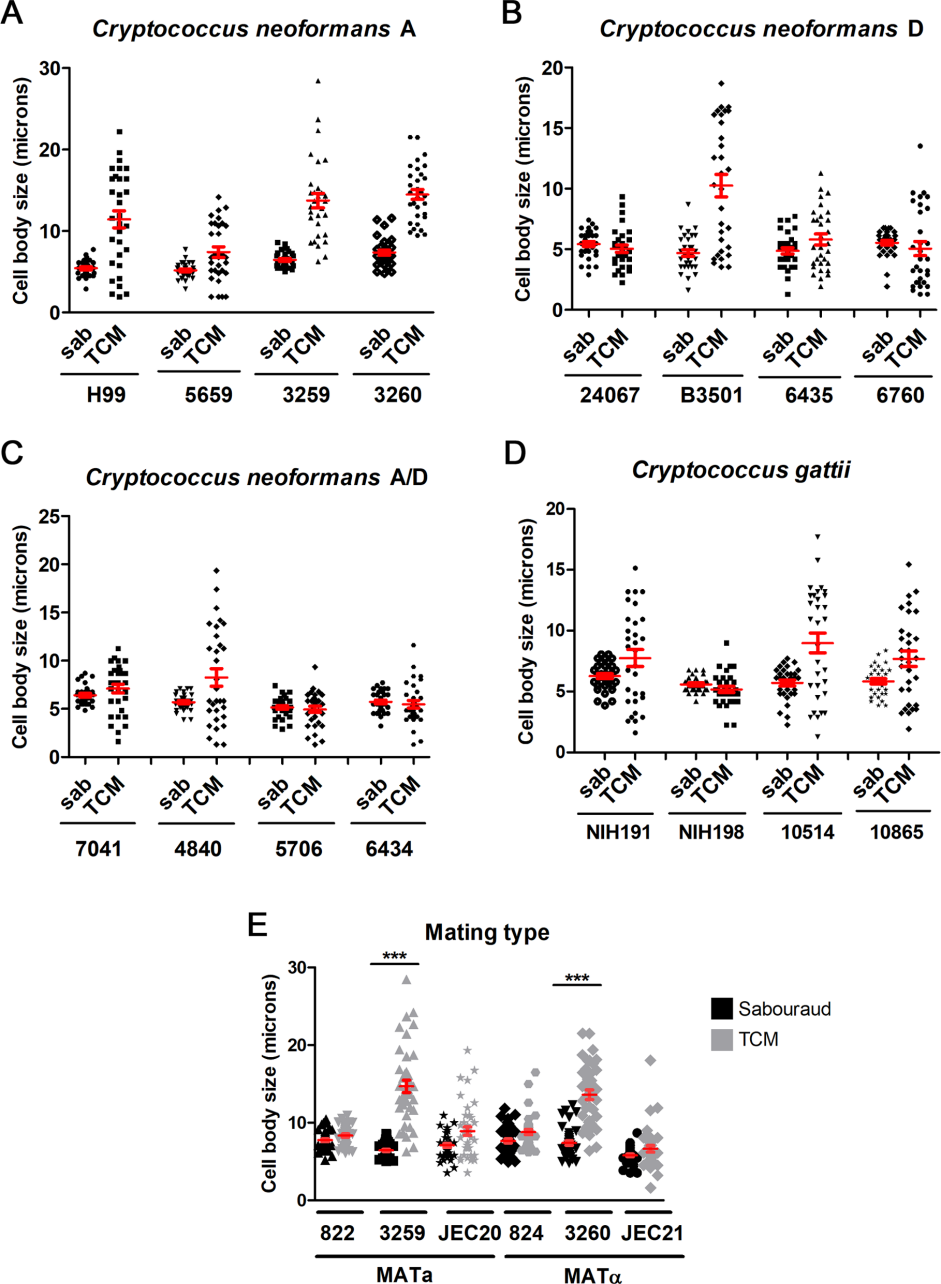
Titan-like cell induction in different strains. Four strains of each species (serotypes) were inoculated into Sabouraud (black symbols) or TCM (grey symbols) at a density of 10^4^ cells/mL and grown overnight at 37°C with CO_2._ (A) *C*. *neoformans* var. *grubii* (serotype A); (B) *C*. *neoformans* var *neoformans* (serotype D); (C) *C*. *neoformans* A/D; (D) *C*. *gattii*. (E) MATa and MATα strains: NE822/NE824 (serotype D), 3259/3260 (serotype A) and JEC20/JEC21 (serotype D). Cell body size was measured in all cases. The red lines represent the mean and standard error.

*Cryptococcus neoformans* is divided in different serotypes and varieties: variety *grubii* (serotype A), variety *neoformans* (serotype D), and A/D hybrids. It has been proposed that these groups should be divided into different species [37], although recent reports argue against this differentiation and suggest the term of species complexes [38]. We investigated if there was a correlation between the main species complex of the strain and the formation of titan-like cells. As shown in Fig 8A–8C, for each serotype/species complex, there were strains with high and low capacity to induce titan-like cells, which indicates that there was not a direct association between the serotype and cell growth.

We also investigated the behavior of strains from the related species *C*. *gattii*, which can infect immunocompetent patients. As shown in Fig 8D, there were strains that had high and low ability to induce titan-like cells. We next examined if the hyper (CBS10514, R265) and hypovirulent (CBS10865, R272) strains isolated at the Vancouver outbreak [39, 40] formed titan-like cells in a different way. Interestingly, the hypervirulent strain tended to produce more titan-like cells compared to the strain with reduced virulence (Fig 8D). We also tested other *C*. *gattii* strains (NIH 191 and NIH198), which also presented differences in their capacity in producing titan-like cells. In summary, the many inter-strain differences in the capability to form titan-like cells did not allow associating this ability to the serotype/genotype of the isolates.

Coinfection with a and α strains results in a higher proportion of titan cells in the lungs [20], so we investigated whether strains from different mating type had different ability to form titan-like cells. We studied three pair of strains with different mating type JEC20/JEC21, NE822/NE824 and 3259/3260 (KN99), and we found that the pair 3259/3260 increased the size of the cell body in the TCM medium compared to the Sabouraud significantly (p<0.05, Fig 8E). In NE822/NE824 and JEC20/JEC21 pairs, there was a small increase in cell size in TCM and we observed a small amount of titan-like cells in this medium although this difference was not statistically significant. These results indicate that titan-like cell formation can occur *in vitro* independently of the mating type of the strains. However, co-incubation of both mating types did not result in a higher proportion of titan cells in TCM (S2 Fig).

### Titan-like cell formation in different mutants

**Fig 9:**
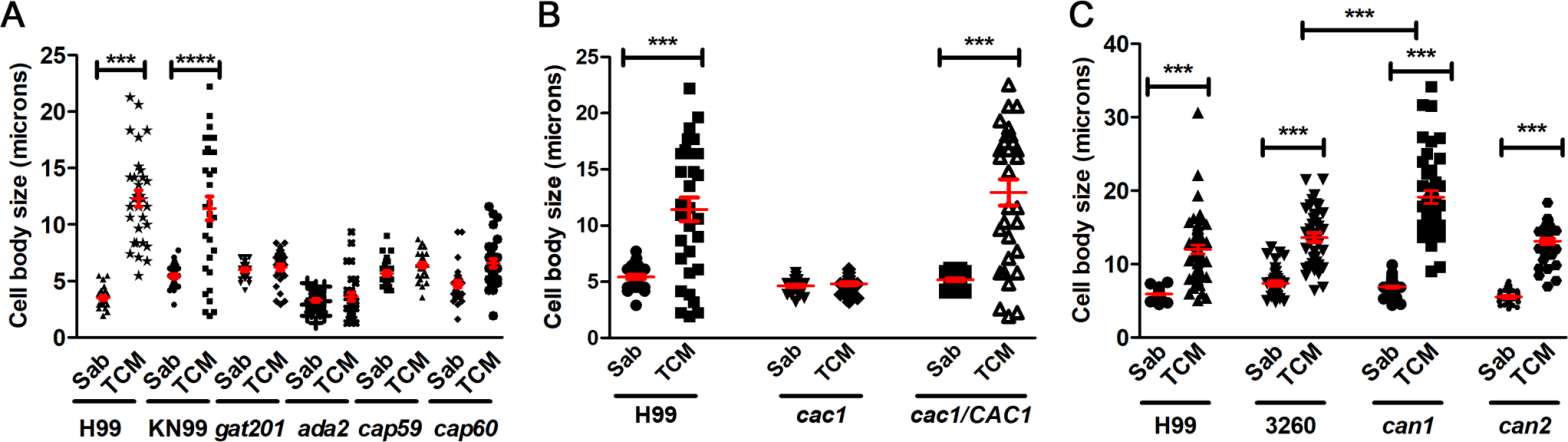
Titan-like cell formation in different mutants. (A) Cells from the capsule deficient mutants *gat201*, *cap59*, *cap60* and *ada2* (H99 background) were inoculated in Sabouraud and TCM medium at a density of 10^4^ cells/mL in 96-well plates. Cell body size was determined after overnight incubation at 37°C with CO_2._ (B) Role of adenylate cyclase on cell growth of *C*. *neoformans*. Cells from the wild-type strain H99, the adenylate cyclase mutant (*cac1*) and the reconstituted strain (*cac1/CAC1*) were incubated as described above. (C) Role of carbonic anhydrases on cell growth. Cells from the wild type strain H99 and KN99 (3260) and the *can1* and *can2* mutants were used for this purpose. Cell body size was determined after overnight incubation at 37°C with CO_2_ in all cases_._ The asterisks indicate significant differences (see M&M). The red lines represent the mean and standard error.

To identify genes that play an important role in the formation of titan-like cells, we studied the phenotype of mutants with problems to induce capsule growth, such as *gat201* or *ada2* [41, 42] or acapsular strains (*cap59* and *cap60*). As shown in Fig 9A, none of these mutants induced cellular enlargement in TCM.

Titan cell formation is regulated by the cAMP pathway [21, 27]. Moreover, the CO_2_ activates adenylate cyclase [43, 44]. For this reason, we investigated the formation of these cells in *cac1* mutant (which lack the enzyme adenylate cyclase) and in the reconstituted strain *cac1/CAC1*. As shown in Fig 9B, the *cac1* mutant was defective to produce cellular enlargement, whereas the reconstituted strain produced titan-like cells as the wild type.

CO_2_ is transformed into HCO_3_^-^ by the action of carbonic anhydrases (Can). In *C*. *neoformans*, there are two genes encoding these enzymes (*CAN1* and *CAN2*) [44, 45], being Can2 the most abundant and physiologically active. Since *can2* mutant can only grow in a CO_2_ enriched environment, these strains were maintained always in 5% CO_2_. Deletion of *CAN2* did not have any effect of titan-like cell formation. Strikingly, in the absence of *CAN1*, titan-like cell size was even larger than that observed for the WT strain in TCM (Fig 9C).

### Interaction of titan-like cells with macrophages

**Fig 10:**
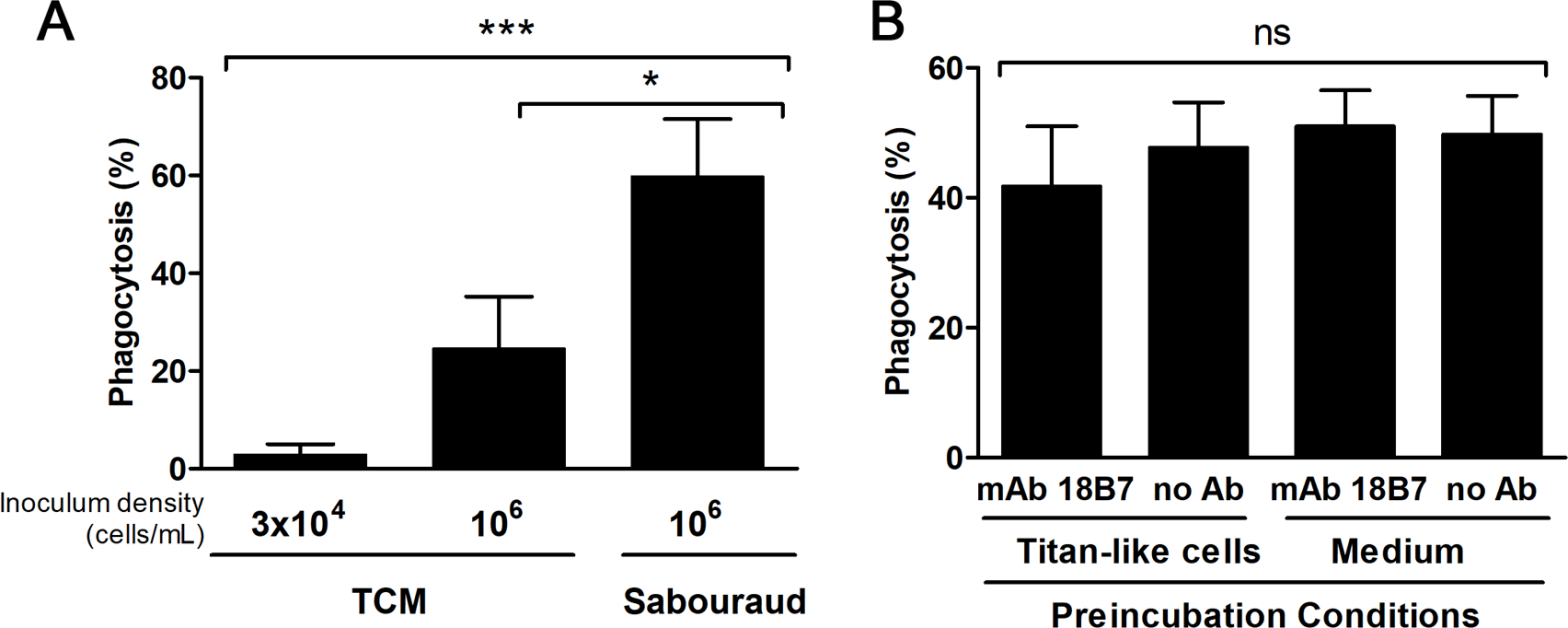
Interaction of titan-like cells with murine-like macrophages. A) Cells from H99-GFP strain were incubated in TCM inoculated at 3x10^4^ cells/mL (titan-like cells), 10^6^ cells/mL (regular cells in TCM), and Sabouraud. Then, phagocytosis experiments with RAW264.7 cells were performed and quantified by flow cytometry as described in material and methods. Statistical differences are highlighted. B) Effect of preincubation of macrophages with titan-like cells on the phagocytosis of regular cells. Titan-like cells were obtained by incubation of *C*. *neoformans* in TCM as described in material and methods, and were exposed to RAW264.7 macrophages at 1:1 ratio. The incubation was performed in media containing the opsonizing mAb 18B7 or in its absence. As control, macrophages were preincubated with growth medium with or without the mAb. The plates were incubated for 1h at 37°C in the presence of 5% CO_2_, and then the cells were washed to remove titan-like cells. Next, *C*. *neoformans* cells (H99-GFP) of regular size grown in Sabouraud were added to the macrophages at 1:1 ratio in the presence of mAb 18B7, and phagocytosis was performed for 1 h at 37°C in CO_2_. Phagocytosis percentage was quantified by flow cytometry as described in M&M. ns: no statistical difference. The experiment was performed in triplicates in three different days.

Titan cells are not phagocytosed *in vivo* [21, 25]. For this reason, we examined the interaction between macrophage-like cell lines and titan-like cells obtained *in vitro*. We compared the phagocytosis of titan-like cells (incubated in TCM inoculated at 3x10^4^ cells/mL), and of cells of regular size obtained in TCM inoculated at 10^6^ cells/mL or in Sabouraud. Phagocytosis was quantified both by microscopic observation and by flow cytometry using a cryptococcal strain that expressed GFP (see Material and Methods). In all cases, we found that titan-like cells obtained *in vitro* were not phagocytosed (Fig 10A and S4 Video). Interestingly, cells of regular size were not equally internalized, since cells cultivated in Sabouraud medium inoculated at high cellular density were more efficiently phagocytosed than the same cells inoculum grown in TCM (S5 and S6 Videos, Fig 10A). This difference was not due to difference in binding to the antibody used as opsonin in this experiment (18B7, S3 Fig), but correlated with the increase in cell size (in particular, due to capsule enlargement) found in cells inoculated in TCM at high density compared to the cells cultivated in Sabouraud (S3 Fig).

It has been shown that titan cells can also prevent phagocytosis *in vivo* of cryptococcal cells of regular size [25], so we tested if titan-like cells induced a similar phenomenon *in vitro*. For this purpose, RAW264.7 macrophages were preincubated with titan-like cells for 1 h with mAb 18B7. As control, parallel samples were incubated only with titan-like cells without mAb or with medium without yeast cells (with or without mAb). After this time, the plate was washed to remove titan-like cells, and H99-GFP cells of normal size cultivated in liquid Sabouraud were added to the macrophages. As shown in Fig 10B, preincubation of the macrophages with titan-like cells did not affect the phagocytosis of regular size yeasts (Fig 10B).

### Analysis of gene expression profile in titan-like cells

To gain insights about the molecular mechanisms involved in titan-like cell formation, we compared their gene expression profile with that of cells of regular size.

**Fig 11:**
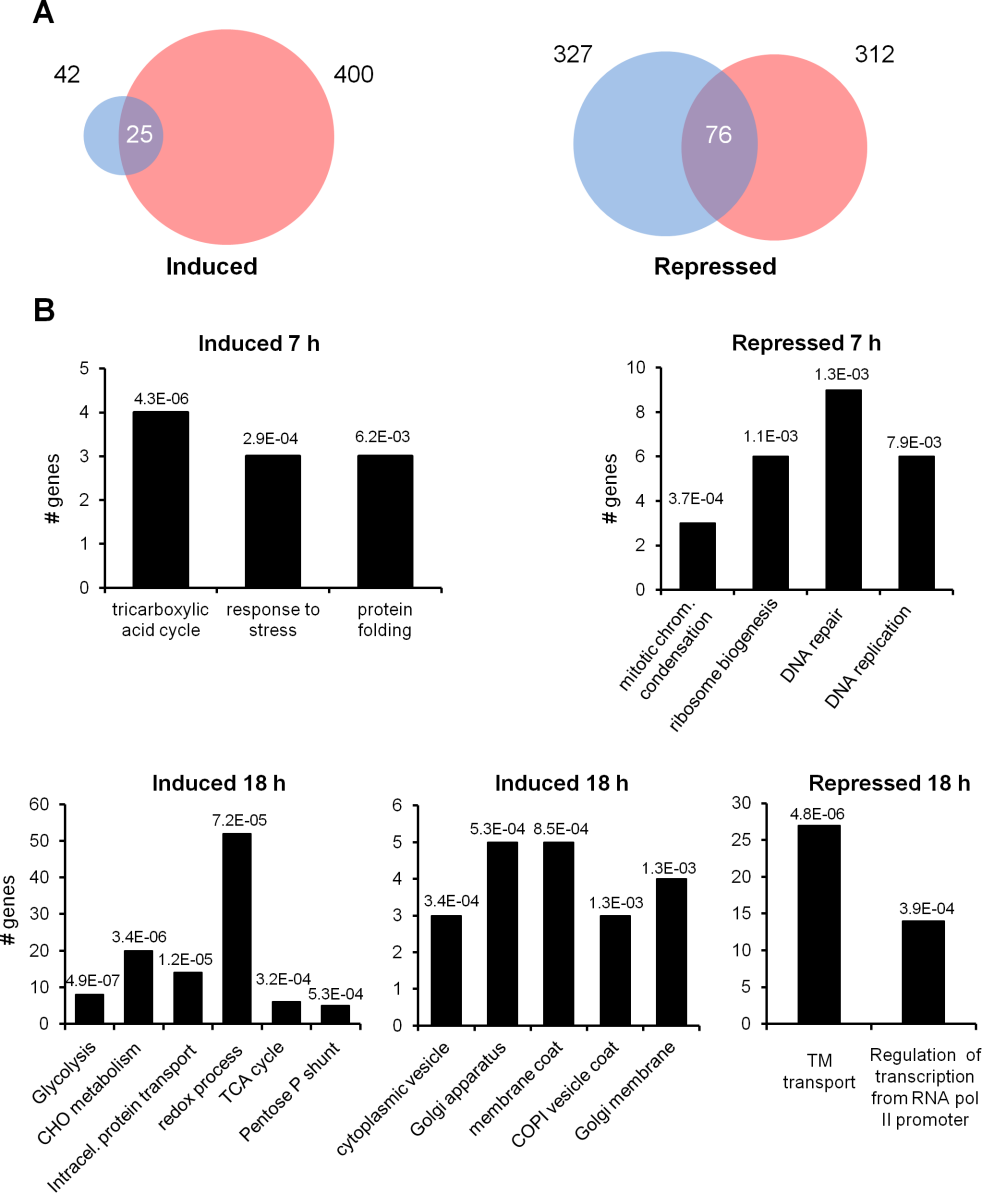
Gene expression changes by RNAseq in titan-like cells obtained *in vitro*. Transcriptomic profiling of the transition to titan-like cells. A) Number of genes induced or repressed after 7 h (blue) or 18 h (red). The number of genes whose expression is modified at both times is denoted at the intersection of the circles. Scale is only approximated. B) Gene Ontology analysis of genes induced or repressed at 7 h (upper panel) or 18 h (lower panel). All classifications correspond to “biological function” terms, except the central graph in the lower panel, which correspond to “cellular components”. Numbers in the histograms denote the *p*-value for each assignment. See Materials and Methods for details.

To this end, cryptococcal cells were inoculated in TCM at low (10^4^ cells/mL, titan-like cells) and high densities (10^6^ cells/mL, regular cells) at 37°C with CO_2_, and total RNA was isolated after 7 and 18 h of incubation. Differences in gene expression were investigated by RNAseq (see material and methods). After mapping the reads and subsequent analysis of differentially expressed genes using the DESeq2 algorithm, we found 42 genes induced at least 1.6-fold in titan-like cells after 7 h and 400 genes induced at least 2-fold after 18 h (Fig 11). A lower expression threshold was set for the 7h data to carry out a comparative GO analysis. Interestingly, the number of repressed genes (after 7h) was relatively high (327) and did not increase after 18 h (312).

Gene Ontology analysis of genes induced at 7 h revealed a moderate increase in genes involved in the tricarboxylic acid (TCA)/glyoxylate cycle and in response to stress and protein folding. The effect of the transition was more evident after 18 h (Fig 11B and Table 1), with the induction of numerous genes involved in carbohydrate metabolism. This included diverse genes related to glycolysis, such as CNAG_03769 and CNAG_05480, encoding two isoforms of hexokinase, CNAG_03916 (glucose-6-phosphate isomerase), CNAG_04676 (6-phosphofructokinase), CNAG_06770 (fructose-bisphosphate aldolase 1), CNAG_02035 (triose-phosphate isomerase) and two isoforms of enolase (CNAG_03072 and CNAG_06868). The TCA cycle was also affected, with induction of mitochondrial citrate synthase (CNAG_00061), E1 and E2 components of ketoglutarate deshydrogenase (CNAG_03674 and CNAG_03596), the two subunits of isocitrate dehydrogenase (CNAG_07363 and CNAG_07851), and CNAG_05653, encoding malate synthase. In addition, genes belonging to the oxidative segment of the pentose-phosphate shunt were also induced, such as glucose-6-phosphate dehydrogenase (CNAG_03245), 6-phosphogluconolactonase (CNAG_02133), and two isoforms of 6-phosphogluconate dehydrogenase (CNAG_04099 and CNAG_07561). Finally, genes involved in trehalose metabolism, such as CNAG_03113, CNAG_03765, and CNAG_05292, encoding a trehalose phosphatase and two trehalose synthases, respectively, were upregulated.

**Table 1:** Genes that were significantly overexpressed (fold>2 with P<0.05) at 7 or 18 h in titan-like cells were subjected to Gene Ontology analysis. For unassigned genes, additional Blastp comparisons against fungal data bases were carried out. T: Titan-like cells (cells incubated in TCM at 10^4^ cells/mL); S (cells of regular size, incubated in TCM at 10^6^ cells/mL).

			7h		18 h	
Process	Gene ID	Description	T/S ratio	*p*-val.	T/S ratio				*p*-val.
Carbohydrate Metabolism						
*Glycolysis (p-value*: *4*.*9E-07)*						
	CNAG_03769	hexokinase	**0.7**		**2.9**	2.1E-05
	CNAG_05480	hexokinase, variant	**1.0**	1.9E-03	**2.6**	2.5E-03
	CNAG_03916	glucose-6-phosphate isomerase	**0.9**	3.6E-02	**2.7**	1.5E-04
	CNAG_04676	6-phosphofructokinase	**1.2**	6.0E-03	**2.1**	2.6E-02
	CNAG_06770	fructose-bisphosphate aldolase 1	**0.8**		**2.0**	2.3E-02
	CNAG_02035	triose-phosphate isomerase	**0.8**	2.3E-02	**2.1**	1.4E-02
	CNAG_03072	enolase (*ENO1*)	**1.3**	2.6E-04	**2.2**	6.5E-03
	CNAG_06868	phosphopyruvate hydratase (*ENO2*)	**0.4**		**3.2**	3.3E-02
*TCA cycle (p-value*: *3*.*2E-04)*						
	CNAG_00061	citrate synthase, mitochondrial	**1.3**	2.5E-03	**2.4**	1.4E-02
	CNAG_03674	oxoglutarate deHase, E1 component	**0.9**	3.3E-02	**2.3**	4.4E-03
	CNAG_03596	2-oxoglutarate deHase E2 component	**0.6**		**2.0**	2.4E-02
	CNAG_07363	isocitrate deHase, NAD-dependent	**1.0**	3.4E-03	**2.8**	2.0E-03
	CNAG_07851	isocitrate deHase, NAD-dependent	**2.1**	1.8E-09	**2.7**	5.7E-04
	CNAG_05653	malate synthase A (MLS1)	**1.8**	2.4E-05	**7.8**	1.7E-11
*Pentose-phosphate shunt (p-value*: *5*.*3E-04)*						
	CNAG_03245	glucose-6-phosphate deHase	**0.6**		**2.8**	3.2E-04
	CNAG_02133	6-phosphogluconolactonase	**0.6**		**2.6**	8.7E-04
	CNAG_01492	hypothetical 6-phosphogluconate deHase	**0.5**		**2.7**	1.0E-03
	CNAG_04099	6-phosphogluconate deHase, decarboxylating	**1.2**	1.2E-02	**3.3**	3.8E-05
	CNAG_07561	6-phosphogluconate deHase, decarboxylating 1	**0.5**		**2.3**	8.9E-03
*Others*						
	CNAG_06313	phosphoglucomutase	**1.2**	4.0E-04	**2.7**	1.9E-04
	CNAG_06923	phosphoketolase	**0.8**		**3.0**	1.6E-04
	CNAG_01155	glycerol kinase	**1.3**	6.6E-05	**4.5**	2.4E-06
	CNAG_03113	trehalose synthase	**1.1**	3.7E-04	**3.7**	3.2E-03
	CNAG_03765	trehalose-phosphatase *(TPS2)*	**0.8**		**3.1**	4.9E-03
	CNAG_05292	α,α-trehalose-phosphate synthase *(TPS1)*	**0.5**		**2.7**	2.4E-03
	CNAG_00373	glucan 1,3-β-glucosidase	**0.3**		**2.0**	4.7E-02
	CNAG_05138	glucan 1,3-β-glucosidase	**0.7**		**2.9**	7.8E-03
	CNAG_05652	glucan 1,3-β-glucosidase, putative	**0.7**		**2.3**	2.3E-02
	CNAG_04033	α1,3-glucosidase	**0.6**		**2.5**	4.8E-03
	CNAG_00393	1,4-α-glucan-branching enzyme	**0.4**		**2.1**	2.6E-02
	CNAG_04879	glycogen debranching enzyme	**0.9**	2.2E-02	**3.4**	2.8E-05
	CNAG_01164	glutamine-fructose-6-phosphate transaminase	**0.9**		**2.2**	1.6E-02
	CNAG_01239	chitin deacetylase	**1.5**		**3.3**	1.9E-02
	CNAG_02445	phosphoacetylglucosamine mutase	**0.8**		**2.5**	2.5E-02
	CNAG_06098	glucosamine-6-phosphate deaminase	**0.9**	2.1E-02	**2.4**	6.4E-03
	CNAG_06659	hexosaminidase	**0.7**		**6.2**	2.4E-06
Intracellular protein transport (p-value: 1.2E-05)						
*COPI and COPII vesicles*						
	CNAG_03554	coatomer, subunit α (*COP1*, *SEC33*, *RET1)*	**0.9**	2.2E-02	**2.1**	6.0E-03
	CNAG_03299	coatomer beta subunit (*SEC26*)	**1.2**	1.1E-03	**2.4**	7.8E-03
	CNAG_04074	coatomer beta' subunit (*SEC27*)	**1.2**	9.5E-03	**2.2**	1.2E-02
	CNAG_01274	coatomer subunit gamma (*SEC21*)	**1.1**	1.0E-03	**2.1**	2.3E-02
	CNAG_01211	putative coatomer subunit epsilon (*SEC28*)	**0.7**		**2.0**	1.5E-02
	CNAG_06773	protein transporter SEC24 *(SEC24*)	**1.2**	2.9E-02	**2.8**	5.5E-03
	CNAG_04803	protein transporter SEC31 (*SEC31*)	**1.3**	5.6E-03	**2.7**	7.9E-03
	CNAG_00148	COPII coat assembly protein Sec16	**1.1**	1.8E-02	**2.8**	6.1E-03
*clathrin-dependent transport from the TNG to endosomes*						
	CNAG_07318	AP-1 complex subunit gamma-1 (*APL4*)	**0.7**		**2.1**	2.4E-02
	CNAG_00977	ADP-ribosylation factor-binding protein (GGA2)	**0.8**		**2.0**	2.4E-02
	CNAG_04499	clathrin light chain	**0.8**		**2.2**	8.0E-03
	CNAG_04904	clathrin heavy chain	**1.0**	2.5E-03	**2.3**	5.6E-03
Others						
	CNAG_01653	cytokine inducing-glycoprotein Cig1	**1.0**	1.4E-02	**3.5**	2.0E-03
	CNAG_00815	Siderophore-iron transporter	**1.2**	1.1E-03	**4.4**	6.3E-11
	CNAG_02500	calnexin	**3.1**	1.0E-12	**3.6**	2.0E-05

Diverse genes encoding closely related enzymes involved in amino sugar metabolism were also induced, including glutamine-fructose-6-phosphate transaminase (CNAG_01164), phosphoacetylglucosamine mutase (CNAG_02445), glucosamine-6-phosphate deaminase (CNAG_06098), and N-acetylglucosamine-6-phosphate deacetylase (CNAG_06098), plus CNAG_02355, coding for a UDP-xylose/UDP-N-acetylglucosamine transporter. The expression of two additional genes related to chitin degradation (CNAG_01239, encoding chitin deacetylase, and CNAG_06659, hexosaminidase), was also increased.

Gene Ontology analysis did not report any significantly enriched category for the set of gene repressed at 7h when the Benjamini-Hochberg adjustment method was used. However, when a non-adjusted search was conducted, we found several genes related to ribosome biogenesis, DNA repair and DNA replication. In particular, three genes (CNAG_00681, CNAG_01959, and CNAG_03148) involved in the nuclear condensin complex, required for establishment and maintenance of chromosome condensation, and 3 out of 4 components of the GINS complex (CNAG_03374, CNAG_02884, and CNAG_04682) which participates in both initiation and elongation of DNA replication. Similarly, the expression of two cyclin-encoding genes (CNAG_03385 and CNAG_00442) belonging to the G1-phase Pcl1,2 family, was clearly repressed (Table 2). These assignations were confirmed by exploring the FunCat classification ontology. Analysis of the repressed genes after 18 h incubation yielded two main GO categories: genes related to transmembrane transport and to the regulation of RNA polymerase II promoters. In the former category there were included diverse sugar transporters (CNAG_01862, CNAG_02733, CNAG_05662, CNAG_06292, and CNAG_06932), plus a likely glycerol proton symporter similar to budding yeast Stl1 (CNAG_04784). The latter category contained 14 putative transcription factors, most of them belonging to the zinc finger transcriptional activator family. However, in most cases their sequence similarity with previously characterized transcription factors was rather low, thus precluding a tentative functional assignment.

**Table 2:** Genes significantly repressed (>2.0-fold and p<0.05) at 7 or 18h during the formation of titan-like cells. Functional assignments were based on Gene Ontology. For unassigned genes, additional Blastp comparisons against fungal data bases were carried out. T: Titan-like cells (cells incubated in TCM at 10^4^ cells/mL); S (cells of regular size, incubated in TCM at 10^6^ cells/mL).

			7 h		18 h	
Process	Gene ID	Description	T/S ratio	*p*-val.	T/S ratio	*p*-val.
*DNA Replication adn DNA Repair (p-value*: *1*.*3E-03)*						
	CNAG_00680	kinetochore protein Nuf2, variant	**0.1**	3.6E-04	**0.4**	6.9E-03
	CNAG_00681	condensin complex subunit 3, variant	**0.2**	6.7E-03	**0.6**	
	CNAG_00991	flap endonuclease 1	**0.1**	2.4E-03	**0.7**	
	CNAG_01144	replication factor A1	**0.3**	4.1E-02	**0.8**	
	CNAG_01430	hypothetical protein	**0.1**	2.8E-03	**0.6**	
	CNAG_01916	DNA mismatch repair protein Msh6	**0.3**	4.8E-02	**0.6**	
	CNAG_01959	condensin complex subunit 1	**0.2**	1.3E-02	**0.4**	6.6E-03
	CNAG_02491	nuclear protein	**0.1**	3.4E-02	**0.2**	2.4E-03
	CNAG_02884	DNA replication complex GINS protein Psf2	**0.2**	2.8E-02	**0.7**	
	CNAG_02963	anaphase-promoting complex subunit 8	**0.1**	2.6E-02	**0.4**	
	CNAG_03148	nuclear condensin complex protein	**0.2**	2.5E-02	**0.5**	4.6E-02
	CNAG_04231	hypothetical protein	**0.2**	3.9E-02	**0.5**	
	CNAG_04682	DNA replication complex GINS protein Psf3	**0.1**	6.5E-04	**0.2**	4.5E-03
	CNAG_05177	DNA polymerase kappa subunit	**0.1**	6.0E-04	**0.3**	3.4E-03
	CNAG_05468	AP endonuclease 1	**0.2**	3.2E-02	**0.4**	
	CNAG_05891	TDG/mug DNA glycosylase	**0.2**	2.9E-02	**0.8**	
	CNAG_06273	hypothetical protein	**0.2**	2.8E-02	**0.5**	
	CNAG_06588	hypothetical protein, variant	**0.2**	3.9E-02	**0.5**	
	CNAG_06634	DNA polymerase epsilon subunit B	**0.2**	1.5E-02	**0.5**	
	CNAG_06793	exonuclease 1	**0.2**	2.6E-02	**0.6**	
	CNAG_07657	U3 small nucleolar RNA-associated protein 19	**0.2**	4.6E-02	**0.5**	
*Ribosome biogenesis (p-value*: *1*.*1E-03)*									
	CNAG_01104	ribosomal RNA-processing protein 8 (*RRP8*)	**0.2**	7.3E-04	**0.5**	
	CNAG_01550	pre-rRNA-processing protein (*TSR3*)	**0.1**	6.1E-04	**0.5**	
	CNAG_01715	ribosome biogenesis protein (*BMS1*)	**0.2**	1.2E-02	**0.7**	
	CNAG_01812	large subunit ribosomal protein L24e	**0.2**	4.8E-02	**0.4**	
	CNAG_02672	nonsense-mediated mRNA decay 3 (*NMD3*)	**0.2**	2.5E-02	**0.5**	
	CNAG_03724	ribosomal RNA methyltransferase Nop2	**0.3**	3.3E-02	**0.5**	
	CNAG_03954	ribosomal RNA-processing protein 17	**0.2**	1.9E-02	**0.4**	
	CNAG_04259	H/ACA ribonucleoprotein complex subunit 1	**0.2**	4.5E-02	**1.0**	
*Transmembrane transport (p-value*: *4*.*8E-6)*									
	CNAG_00726	Allantoate permease *(DAL5)*	**0.0**		**0.0**	3.4E-02
	CNAG_00898	multidrug efflux pump, YOR378W-related	**0.1**		**0.0**	4.9E-03
	CNAG_00904	aflatoxin efflux pump Aflt	**0.1**		**0.0**	7.5E-03
	CNAG_01118	putative dicarboxylic amino acid permease	**0.1**	1.0E-03	**0.2**	4.2E-02
	CNAG_01545	hypothetical protein, variant	**0.2**		**0.3**	3.3E-02
	CNAG_01862	hexose transporter (*STL1*, *HXT8*, *HXT13*,. family)	**0.3**		**0.2**	4.7E-02
	CNAG_02595	hypothetical protein, variant	**0.1**	2.7E-02	**0.2**	1.5E-02
	CNAG_02733	hexose transporter (*STL1*, *HXT8*, *HXT13*,. family)	**0.3**		**0.0**	6.9E-03
	CNAG_02777	Pi transporter PHO84	**0.4**		**0.5**	1.4E-02
	CNAG_04567	putative TPO-related spermine transporter	**0.2**		**0.3**	2.8E-02
	CNAG_04597	hypothetical protein	**0.2**		**0.3**	1.8E-02
	CNAG_04784	Sugar transporter (*STL1*, *SNF3* family)	**0.1**		**0.2**	3.3E-02
	CNAG_04794	spermine transporter (*TPO2*, *TPO3* family)	**0.2**	5.3E-04	**0.5**	4.4E-02
	CNAG_04818	MFS monocarboxylate transporter, putative	**0.1**	2.2E-02	**0.2**	2.4E-02
	CNAG_05662	hexose transporter (*HXT8*, *HXT13*, *HXT17* family)	**0.1**	1.2E-02	**0.3**	2.2E-02
	CNAG_05867	L-fucose permease, putative	**0.2**		**0.2**	1.1E-05
	CNAG_05992	Allantoate permease	**0.1**		**0.0**	1.9E-02
	CNAG_06102	ADP,ATP carrier protein, variant 1	**0.1**	6.6E-05	**0.2**	7.5E-03
	CNAG_06292	sugar transporter	**0.1**		**0.2**	2.5E-02
	CNAG_06557	N-acetylglucosamine-specific transporter	**0.6**		**0.0**	3.2E-03
	CNAG_06561	allantoate transporter	**0.0**		**0.0**	2.6E-02
	CNAG_06758	multidrug efflux pump, YOR378W-related	**0.3**		**0.2**	2.9E-04
	CNAG_06932	sugar transporter (*STL1*, *HXT17*, *HXT13* family)	**0.2**		**0.2**	6.7E-03
	CNAG_07449	General amino acid permease (*AGP2*)	**0.1**		**0.2**	2.8E-02

Interestingly, several genes encoding proteins involved in Golgi-related protein traffic were induced, specifically five out of the seven components of the COPI-coated vesicles, also known as coatomer (α, β, β', γ, and ε, encoded by CNAG_03554, CNAG_03299, CNAG_04074, CNAG_01274, and CNAG_01211, respectively). COPI-coated vesicles are found associated with Golgi membranes, are involved in Golgi to endoplasmic reticulum (retrograde) vesicle transport, and also likely in intra-Golgi transport. The expression of components of the COPII coat, such as CNAG_06773 (*SEC24*) and CNAG_04803 (*SEC31*), as well as that of the interacting protein CNAG_00148 (*SEC16*) also increased. In addition, genes encoding proteins required for cargo-selective, clathrin-dependent transport from the TNG to endosomes in yeast were induced. These are CNAG_00977, encoding a likely homolog of the budding yeast monomeric clathrin adaptor proteins *GGA1* or *GGA2*, CNAG_07318, encoding the AP-1 complex subunit gamma-1, CNAG_06564, coding for an AP-1 interacting protein, or the genes coding for clathrin light and heavy chains (CNAG_04499 and CNAG_04904).

**Fig 12:**
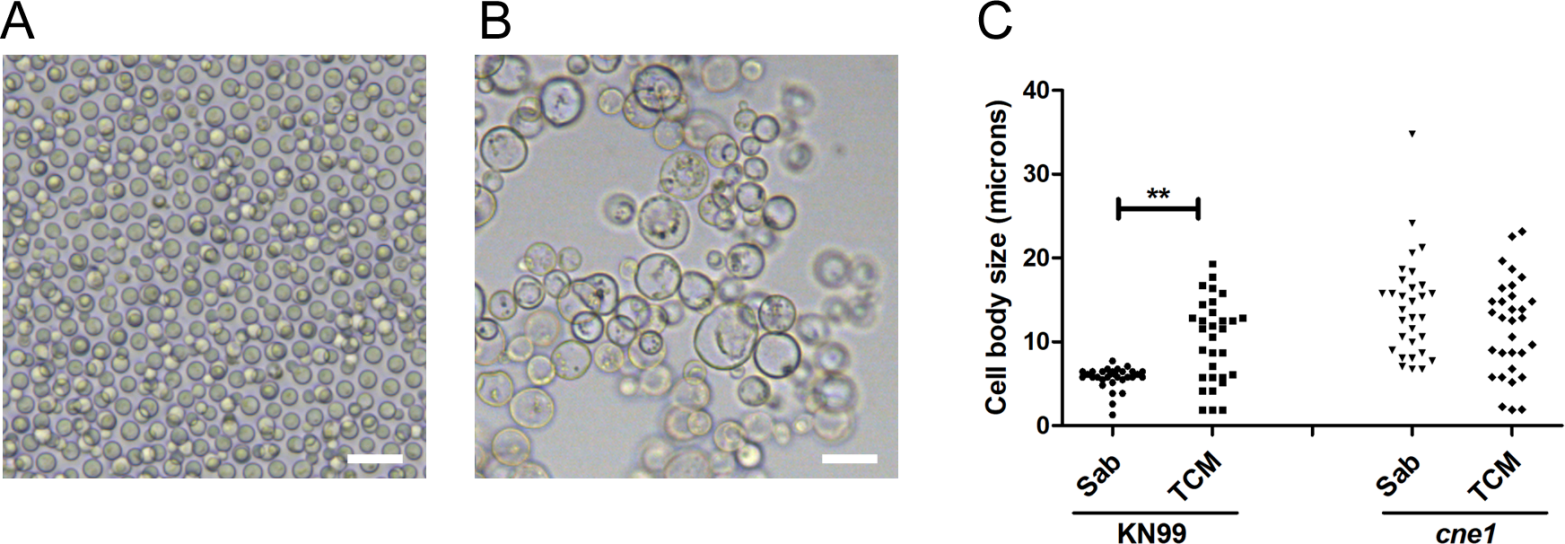
Phenotype of the mutant lacking calnexin (*cne1*). Pictures of the WT (KN99 strain,A) and a *cne1* mutant (lacking calnexin, CNAG_02500, B) in liquid Sabouraud medium. The scale bars denote 20 μm. (C) Measurement of cell body size of the WT strain (KN99) and the mutant lacking calnexin. The cells were incubated in liquid Sabouraud at 30°C or TCM (overnight) at 37°C with 5% CO_2_.

One of the genes clearly overexpressed at both 7 and 24 h encoded Calnexin (CNAG_02500), which is a chaperone located at the ER that contributes to the proper folding of glycoproteins. To test if this protein had any effect on titan cell formation, we obtained the mutant and investigated its phenotype. As shown in Fig 12, in the absence of calnexin, the cells had significantly larger size in regular rich medium compared to the wild type strain (Fig 12), and no change was detected when the cells were transferred to TCM. This result confirmed that calnexin was involved in processes that regulate cell size in *C*. *neoformans*.

**Fig 13:**
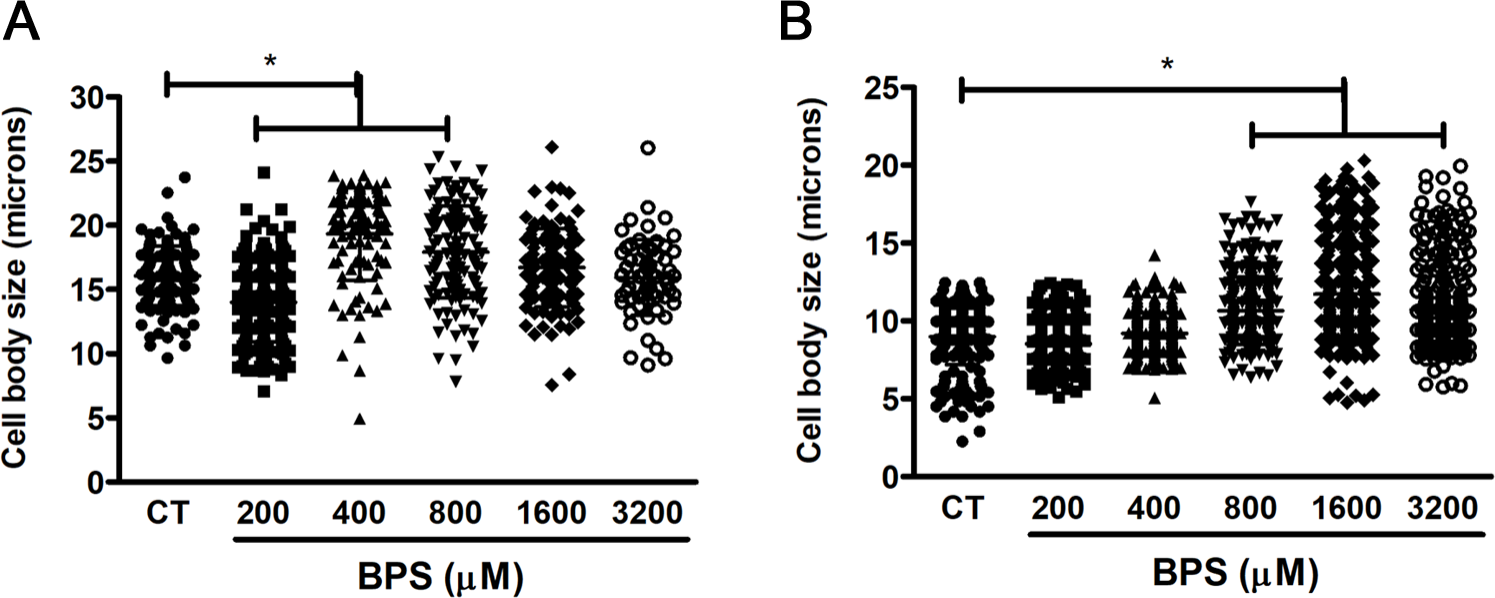
Effect of iron chelation on cryptococcal morphology. The effect of iron chelation on cell body size was observed by the supplementation of TCM with the iron chelator Bathophenanthroline disulfonic acid (BPS) in TCM cultures inoculated at 10^4^ cells/mL cultures (A) and 10^6^ cells/mL cultures (B). The cells were incubated at 37°C overnight with 5% CO_2_ without shaking. CT, control cells without BPS.

We also noticed that some genes related to iron metabolism were also induced in titan-like cells compared to cells of regular size. One of them (CNAG_01653) encoded Cig1, (initially annotated as cytokine inducing glycoprotein), which is involved in iron uptake from heme groups and is overexpressed during iron limitation conditions [46, 47]. Iron is an important element required by yeasts, and in *C*. *neoformans*, its limitation also induces capsular enlargement. For this reason, we investigated whether iron concentration had any effect on titan-like cell formation. As shown in Fig 13, depletion of iron using the chelator BPS induced the appearance of cells of large size when the cultures were inoculated at high cell densities (10^6^ cells/mL), suggesting that iron limitation also contributes to cellular enlargement.

## Discussion

*Cryptococcus neoformans* is an exceptional model to understand mechanisms induced by pathogenic fungi to adapt to the host and cause disease. Some of the most important changes are related to changes in cell size, which can occur by growth of the capsule, or growth of both the cell body and capsule. In this last case, *C*. *neoformans* induces a specific cell type that has been denominated titan cells [20, 21]. At the moment, only the cAMP-dependent and the mating pathways have been described as relevant in titan cell formation [20, 21, 27]. It has been shown that increased expression of PKA induces cell enlargement in *C*. *neoformans* and the ploidy of the cells [48, 49]. One of the limitations to study titan cells is the difficulty of reproducing this phenomenon *in vitro*. Originally, we described that a small proportion of titan cells could be observed in minimal media [21]. Then, it was described that these cells appear in the presence of phospholipids [28]. In this work, we have defined a medium in which we have consistently replicated this phenomenon *in vitro*. This medium has several characteristics: limitation of nutrients at neutral pH, inclusion of mammalian serum and a CO_2_ enriched atmosphere. In these conditions, we could obtain a high proportion of cells of a size around 30 μm (capsule included). This diameter is smaller than the average size measured in cells isolated from mouse lungs [11, 20, 22], where average size is around 40–50 μm, so we argue that in our TCM we could be examining the initial steps of this morphological transition. It is possible that *in vitro* the cells do not reach the same size than cells obtained from *in vivo* infections because the nutrients of the medium or the factors that favor this morphological change are consumed. Instead, *in vivo* infections are maintained for a longer time, with a stable nutrient concentration allowing cells to reach significantly larger sizes. Despite this limitation, the availability of *in vitro* conditions that, at least in part mimic titan cell formation is a key contribution to understand the biology of these cells.

In general, our results indicate that titan-like cell formation is induced by multiple factors, being some of them necessary, but not sufficient, to trigger the transition. This is the case of serum which only induced cell growth under nutrient limiting conditions. These results indicate that titan-like cells are formed in response to some elements of the host present in the serum in the context of the stress produced by the limitation of nutrients in the medium. These findings allow the dissection of the intracellular pathways that are triggered during cellular growth.

It has been described that serum and the fraction of polar lipids induce cell enlargement in *C*. *neoformans*. Our results agree with previous findings [28], because polar lipids were also able to induce the formation of titan cells in TCM. The most plausible mechanism for this effect is that phospholipids are degraded by phospholipase C, which produces diacylglycerol (DG), or by phospholipase B, which produces arachidonic acid (AA) [50]. DG activates human as well as *C*. *neoformans* PKC [34]. To investigate the role of this signaling pathway, we blocked the activity of this kinase with several pharmacological inhibitors, and found that both staurosporine and calphostine C had a dramatic effect on titan-like cell formation. This protein participates in multiples processes, such as maintenance of the integrity of the cell wall [51–53] and polarized growth [54]. Mutants lacking the *PKC1* gene present many cellular alterations, such as osmotic instability and susceptibility to temperature [35]. We tried to evaluate the effect of these inhibitors at a lower temperature and in the presence of sorbitol in an attempt to overcome the cellular defects associated with the absence of Pkc1. For this reason, further studies using mutants affected in signaling pathway components are required to understand in detail how this pathway participates in titan-like cell formation.

We also observed that other important factor for titan-like cell formation *in vitro* is CO_2_. The capsule is induced in response to CO_2_ concentrations present in the host [55], which is consistent with this factor also favoring the growth of the cell body. Carbonic anhydrase converts CO_2_ into HCO_3_^-^, which activates adenylate cyclase [44, 56], so our results are consistent with the hypothesis that CO_2_ induces cell growth through the cAMP pathway. In *C*. *neoformans*, there are two carbonic anhydrases encoding genes, *CAN1* and *CAN2*, with *CAN2* being the most important [44]. Interestingly, deletion of *CAN1* resulted in an enhancement of the production of titan-like cells. Although we do not know the molecular mechanism for this phenomenon, we postulate that in the absence of Can1, there might be a compensatory overexpression of Can2 that could induce the hyperactivation of the cAMP pathway.

We also observed that subinhibitory concentrations of azide have a modest, but reproducible positive effect on titan-like cell development. We argue that a partial inhibition of the respiratory chain can trigger a stress signal that results in a stop of the cell cycle and allows cellular size increase. In this regard, it could be assumed that a limitation in the respiratory capacity might lead the organism to generate energy, at least in part, by fermentative metabolism, a situation that has been associated to increased PKA activity in many fungi [57]. Although *C*. *neoformans* is mainly a respiratory yeast, it has been shown that it can produce both ethanol and acetate *in vitro* and *in vivo* [58, 59], so a similar activation of the PKA activity could also occur in these conditions. However, the effect of the mitochondrial inhibitor was not significant in the presence other factors (in particular, serum and CO_2_). Since CO_2_ is an activator of the adenylate cyclase, it is reasonable to argue that in these conditions the effect of azide would be not significant.

One of the most striking results of this work is the effect of cell density on the formation of titan-like cells, suggesting that this transition is regulated by *quorum sensing* (QS) phenomena. In this way, a higher cell density results in a higher concentration of released molecules and greater effect on the cells [29, 60, 61]. A major QS signal for *C*. *neoformans* is a 11-mer peptide (Qsp1) [31, 32]. We tested the effect of this peptide on titan-like cell formation, and found that it inhibited the transition. Qsp1 promotes cell division and replication. Although further studies are required to understand the molecular mechanism by which Qsp1 regulates this transition it is plausible to propose that, since titan-like cells are formed in the absence of budding, Qsp1 blocks this development due to its positive effect of cell division and replication. The biological role of QS phenomena on titan-like cells is not known. However, it is worth noting that a cell-dose relationship similar to that found here has been described *in vivo*, since a low number of cryptococcal CFUs in the lung correlates with a higher proportion of titan cells [21]. Therefore, it will be necessary to elucidate the role of Qsp1 in the context of lung colonization in the future. However, this peptide does not seem to be the only factor that represses cellular growth at high densities, since *qsp1* mutants form titan-like cells similarly to the wild type strain. In agreement with this notion, *C*. *neoformans* produces QS molecules that are not susceptible to high temperature, proteinase, trypsin, pronase, DNAse, RNAse and glucosidase [30]. These authors also described that farnesol, tyrosol and Qsp1 did not replicate the effects observed with their conditioned media, and demonstrated that pantothenic acid could in part reproduce QS phenomena. In summary, we hypothesize that several QS molecules negatively regulate titan-like cell formation.

The formation of titan cells has been observed in clinical samples [62–64], and despite being a mechanism that confers advantages to *C*. *neoformans* against the host during infection, we observed that this process is not a universal phenotype. Our results showed that there is great variability among different isolates, and not all the strains did form titan-like cells *in vitro*. Our work provides a new way to investigate the genetic differences between strains with high and low capacity to form titan-like cells using genomic approaches. However, the strains of *C*. *neoformans* var. *grubii* (serotype A) exhibited the highest proportion of titan-like cells, suggesting that this serotype has a greater capacity of adaptation to the lung. This result is in agreement with the literature, since this serotype is the one most frequently isolated in infected patients [10], suggesting that there is a correlation between the ability to form titan cells and development of the disease. We believe that this correlation should be confirmed in future clinical studies.

Previous reports demonstrate that during capsule growth, the size of this structure correlates with cell body size [65]. In addition, capsule growth mainly occurs in G1 [66], which is also the cell cycle phase in which the growth of the cell body occurs. However, although titan-like cells increased capsule thickness, the size of this structure was significantly smaller than the one found in titan cells isolated from animals, indicating that the process that occurs *in vitro* is, at least quantitatively, not fully equivalent to the one observed *in vivo*. We argue that the phenomenon that we observed *in vitro* in this work describes the first steps of titan cell formation. In addition, it is reasonable to argue that when the cell body enlarges significantly, there could be a metabolic limitation affecting the growth of the capsule, since its volume increases with the cube value of the radius.

Titan cells contribute to virulence through different mechanisms, such as polarization of Th2-type immune responses [23, 24], replication [26, 67], resistance to oxidative damage [20, 21] and phagocytosis avoidance [21, 25]. In our case, titan-like cells obtained *in vitro* were not phagocytosed, most probably due to their large size. In our case, these cells did not prevent the phagocytosis of cryptococcal cells of regular size, as it is the case in experiments performed *in vivo* [25]. We believe that activation of the macrophages *in vivo* is different from *in vitro*, so in the lungs there could be multiple factors that affect the phagocytic activity of macrophages, explaining why the effect of titan cells *in vivo* on macrophages are not identical from the effects induced *in vitro*. Furthermore, it is also possible that titan cells *in vivo* express virulence factors or epitopes that are not produced *in vitro*. Further studies are required to fully understand the response of immune cells to cryptococcal titan-like cells.

The isolation of titan-like cells *in vitro* opens new perspectives and research lines. For example, we investigated gene expression changes associated with titan cells development. In our approach, we compared cells of different size incubated in the same medium (TCM), so the cells were exposed to the same nutritional environment and inducing factors (CO_2_ and serum). The difference in cell size was obtained due to the different initial cellular density of the cultures (10^4^ vs 10^6^ cells/mL). In this way, our approach would also detect those changes due to quorum sensing phenomena. Despite the cells were incubated in the same medium, we detected changes in many genes involved in metabolism at both times (7 and 18 h). This suggests that the increase in cell size requires a metabolic adaptation that allows the efficient use of extracellular and intracellular sources of nutrients. The induction of many genes involved glycolysis and TCA cycle suggest a higher demand of energy, whereas the increase in the expression most of the components of the oxidative branch of the pentose phosphate shunt (including glucose-6P dehydrogenase, which catalyzes the rate-limiting step) suggest an increased need for NADPH, a requirement for anabolic biosynthetic pathways. Such requirement could be explained by a higher demand for fatty acid synthesis to provide structural components for an increasing membrane surface. Alternatively, it could be considered that induction of a large number of metabolic enzymes would be simply required to maintain its normal concentration in response to a growing cellular volume.

Our results also highlight other processes that might be involved in titan cell formation. In particular, we found that several genes encoding components of COPI and COPII vesicles, which belong to the coatomer complex. These proteins cover the surface of vesicles involved in intracellular protein sorting and early secretory pathway. Interestingly, we found that clathrin- encoding genes were also induced. Although we do not know how these proteins contribute to cryptococcal cellular enlargement, we argue that titan cells might recycle, import and export proteins at a higher rate than regular cells. Interestingly, we also found that the expression of genes encoding some chaperones, such as calnexin, involved in folding of glycosylated proteins increased in titan-like cells, and mutants lacking this protein have an abnormal large size, even in regular rich medium. This result was somehow unexpected, because the expression of this gene increased in titan-like cells. In consequence, we argue that calnexin plays a role, not only in proper folding of glycosylated proteins, but also regulates cell size in conditions of active protein synthesis and replication. In addition, it is possible that it absence results in an stress signal that results in cell cycle changes and G1 arrest, that would produce an increase in cell size too. In this sense, mutants lacking Atg7 (which is part of the ubiquitin-activating enzyme (E1) family and is required for proper authophagy [68]) have also increased cell size and higher DNA content, supporting the idea that titan formation is affected by the trafficking and recycling of intracellular proteins.

Our results indicated that iron depletion from the medium can also regulate the induction of titan-like cells. Interestingly, iron limitation also induces the growth of the cryptococcal capsule [69]. Iron is an important cofactor required for multiple enzymatic activities, so its absence is an important stress signal that results in the expression different acquisition mechanisms, involving siderophores, Cig1 and transporters [70]. Our findings are in agreement with the idea that titan-like cell formation is a response to nutritional stress.

Titan cells isolated from mice are polyploid, so presumably the factors and signaling pathways must alter the normal progression of cell cycle. Interestingly, we found that in titan-like cells, genes related to DNA replication were repressed. In agreement, G1/S cyclins, like Pcl1, were repressed, which suggests that the process requires an elongation of the G1 phase. This result is in agreement with previous findings that demonstrated that *pcl1* mutants have increased capacity to form titan-like cells *in vivo* [27]. In our titan-like cells, we found that they have a higher content of DNA compared to cells of regular size. However, the DNA content of titan-like cells is lower than the content in titan cells isolated from mice, indicating that cell cycle changes that occur *in vitro* are different from *in vivo*.

Finally, we would like to acknowledge that other groups (Dr. Alanio, Pasteur Institute, France, and Dr. Ballou, University of Birmingham, UK) have identified in parallel to our work other conditions in which titan-like cells are formed. We believe that these works together with our findings provide significant advances to the scientific community because they will allow the design of multiple research lines that will facilitate the characterization of the factors and signaling pathways that are involved in titan cell development. The data presented by different groups also indicate that *C*. *neoformans* may induce titan-like cells *in vitro* in response to multiple factors. In addition, the ability to obtain titan cells *in vitro* will also have a positive bioethical impact because we will be able to significantly reduce the number of experimental animals compared to what was required previously to characterize these cells.

## Material and methods

### Strains and media

In most of the experiments, we used *C*. *neoformans* var. *grubii* (serotype A) H99 strain [71], but we also included *C*. *neoformans* var. *neoformans* (*C*. *deneoformans*, serotype D), A/D hybrids and *C*. *gattii*, and different mutants obtained from the library described by Liu and coworkers [41] and from the Fungal Genetic Stock Centre. All strains are described in Table 3. Strains were preserved in Sabouraud medium containing 30% glycerol at -80% and were recovered at 30°C in Sabouraud solid medium (Oxoid LTD, UK).

**Table 3:** List of strains used.

Species	Strain	Reference or Source
***C*. *neoformans*** ***var*. *grubii*** ***(C*. *neoformans*, serotype A)**	H99	[71]
	CL-5659	Mycology Reference Laboratory (CNM)
	3259 KN99 (MATa)	Institut Pasteur
	3260 KN99 (MATα)	Provided by Guilhem Janbon (Institut Pasteur)
	H99-GFP	[77]
	*gat201*	[41]
	*can1* (NE411)	[44]
	*can2* (NE 417)	[44]
	RPC3 (*cac1*)	[82]
	RPC7 (*cac1*::*CAC1*)	[82]
	*cne1*	This work
	*cap60*	Obtained from collection described in [41]
	*qsp1*	[32] obtained from FGSC
	*ada2*	[42] obtained from FGSC
***C*. *neoformans*** ***var*. *neoformans*** ***(C*. *deneoformans*, serotype D*)***	ATCC 24067	American Type Culture Collection
	B3501	[83]
	CL-6435	Mycology Reference Laboratory (CNM)
	CL-6760	Mycology Reference Laboratory (CNM)
	JEC20 (MATa)	[84]
	JEC21 (MATa)	[84]
	NE 822 (MATa)	[85]
	NE 824 (MATα)	[85]
	C536 *(cap59*)	[86]
***C*.*neoformans hybrids*** **(A/D)**	CL-4041	Mycology Reference Laboratory (CNM)
	CL-4840	Mycology Reference Laboratory (CNM)
	CL-5706	Mycology Reference Laboratory (CNM)
	CL6434	Mycology Reference Laboratory (CNM)
***C*. *gattii***	NIH 191	NIH, Bethesda, Maryland
	NIH 198	NIH, Bethesda, Maryland
	CBS-10514 (R265)	[40]
	CBS-10865 (R272)	[40]

For clarity, we have specified the different suggested nomenclatures for the *Cryptococcus* species complex, specifying the varieties, serotypes and species name. In the case of *C*. *gattii*, we preferred to use the classical nomenclature that groups all the genotypes, serotypes and species because we did not include different strains from all these different groups.

In order to delete the *CNE1* (CNAG_2500), a gene a disruption cassette was constructed by overlapping PCR [72] using PCR fragments amplified from either the plasmid pHYG (kindly given by Prof. Jennifer Lodge, Washington University in St Louis, USA) or genomic DNA extracted from the strain KN99α [73].

The *C*. *neoformans* strain KN99α was then transformed by biolistic delivery [74] and transformants were screened on YPD medium containing 200 U ml-1 of hygromycin (Calbiochem). The correct deletion of the gene was tested using primers within and outside the cassette. Finally, the absence of additional ectopic integration of the cassette was checked by Southern blot experiment. Two independent *cne1Δ*::*HYG* strains (NE375 and NE376) were selected for further studies.

The primers used in these PCR experiments are listed below

CNE1seroAex

CCATCTCTTCTTCGGAATCCG

CNE1seroA-5’5

TAGCACTGTGAATCGATCCCG

CNE1seroA-5’3

GTCATAGCTGTTTCCTGGGATGGGATGAATGGAAGACG

MKRrCNE1seroA

CGTCTTCCATTCATCCCATCCCAGGAAACAGCTATGAC

CNE1seroA-3’5

TACAACGTCGTGACTGGGGAGATTCCTGCTGAAGGCTCG

MKRfCNE1seroA

CGAGCCTTCAGCAGGAATCTCCCCAGTCACGACGTTGTA

CNE1seroA-3’3

TGTTACGTTCGACTTGACGCTG

CNE1seroAex2

ACAACGCTTCGACATCTGCAG

The yeasts were routinely grown in liquid Sabouraud medium at 30°C or 37°C with shaking (150 r.p.m.). To induce titan-like cells, strains were grown in a medium that we have defined as Titan Cells Medium (TCM), which is based in the medium described to induce capsule growth (10% Sabouraud buffered at pH 7.3 with 50 mM MOPS, [75]). TCM contains 5% Sabouraud and 5% inactivated fetal calf serum (FCS, Biological Industries) diluted in MOPS 50 mM at pH 7.3 plus 15 μM sodium azide (Sigma Aldrich). Cultures were grown in tissue culture flasks or 96-wells plates at 37°C in an atmosphere enriched with CO_2_ for 18 hours. For iron depletion different concentrations of bathphenanthroline disulfonate (BPS, Sigma Aldrich) were prepared directly in TCM. Cells were then inoculated at 10^4^ or 10^6^ per mL and incubated at 37°C with 5% CO_2_ for 18 hours.

### India Ink staining and measurement of cell size and capsule permeability

To observe and measure the size of the cells, 10 μL of a cell suspension were mixed with a drop of India Ink drop (Remel Bactidrop, Lenexa, Kansas) and observed under a Leica DMI 3000B microscope. Pictures were taken with a Leica DFC 300FX camera using the Leica Application Suite (Leica Microsystems) and processed with Adobe Photoshop 7.0 (San Jose, CA) or ImageJ (https://imagej.nih.gov/ij/) [75]. In some experiments, the permeability index was measured using fluorescently-labeled dextrans as described in [67].

### Infections of mice with *C*. *neoformans* and yeast isolation from lungs

Six to eight weeks-old male mice from C57BL/6J (in house bred at the National Centre for Microbiology) were used in all experiments. The animals were kept in ventilated racks at 22–24°C with proper environmental enrichment (cupboard houses and hollow cylinders).

Yeast cells were incubated overnight in liquid Sabouraud medium at 30°C, centrifuged at 2830 g, washed and suspended in sterile PBS. The cell density was determined using a TC20 cell counter (BioRad) and a suspension of 3.3x10^7^ cells/mL was prepared in sterile PBS. The animals were anesthetized with a mixture of ketamine (Imalgene 1000, 50 mg/Kg) and xylazine (Xilagesic 2%, 20 mg/Kg) and infected intranasally with 30 μL of the yeast suspension (10^6^ cells per mouse) as previously described [23]. Animals were sacrificed after 14 days of infection by exposure to a high CO_2_ enriched environment.

We excised and homogenized the lungs in 10 mL of sterile water using cell strainers (100 μm size pore, BD Falcon) and a 5 mL syringe plunge in Petri plates. This process disrupts the mammalian cells without significantly affecting the integrity of the cryptococcal cells. Then the cell suspension was centrifuged at 2,830 g and washed with sterile water three times to fully break and remove the mouse cells. Finally, the yeasts were suspended in PBS.

### Lipids extraction of fetal bovine serum (FBS)

Polar lipids from serum were obtained as described in [28]. Briefly, aliquots of 1 mL of FBS were shaken with a mixture of chloroform and methanol (2:1) (v:v) for 3 hours at room temperature. The samples were centrifuged for 10 minutes at 2,830 gs for phase partitioning. The upper phase was collected in a 1.5 mL tube and dried during 1 hour in a SpeedVac concentrator. The pellet was suspended in 200 μL of PBS and conserved at 4°C. To evaluate the effect of serum polar lipids on titan-like cell formation, we performed experiments with different amounts of the extraction solution described above (1/40; 1/100 and 1/200 dilution) in 5% Sabouraud buffered at pH 7.3 with 50 mM MOPS and 15 μM sodium azide. As a control, the same medium with PBS was used. In parallel, cells were grown in Sabouraud and TCM as growth control and titan-like cell formation respectively.

To evaluate the role of phosphatidylcholine (Sigma Aldrich), a 20 mM stock of this phospholipid was prepared in EtOH 100%. Then, 1/10 dilutions were done in distilled water to yield 0.1, 0.01 and 0.001 mM (final concentration) in TCM without serum. Same dilutions of EtOH 100% were added to the medium as control.

### Visualization of titan-like cell formation by real-time microscopy

Yeast cells were inoculated in TCM at 10^4^ cells/mL as detailed above. One hundred seventy μL from the yeast suspension were placed in a 96 well plate and incubated at 37°C with 5% CO_2_ under a Leica DMI 4000B microscope. Photographs were taken every 3 min using the 20x objective. The videos generated by the Leica software were exported as AVI documents and processed with ImageJ software. In all cases, the videos were assembled with 12 frames per second, so one second of video corresponds to 36 minutes of real time. Time was included in each frame using the Time Stamper plugin from ImageJ.

### Effect of conditioned media and Qsp1 on titan-like cell formation

Cell suspensions were prepared at a concentration 10^6^ cells/mL in parallel in Sabouraud with 15 μM sodium azide and TCM. Serial 1/10 dilutions were made up to 10^3^ cells/mL. A volume of 170 μL of these suspensions was incubated in a 96 well plate at 37°C overnight with 5% CO_2_ without shaking. The plates were observed with a Leica DMI 3000B microscope. Pictures were taken with a Leica DFC 300FX camera using Leica Mycrosystems software. The cell body diameter of thirty to fifty cells was measured with Adobe Photoshop 7.0.

*Cryptococcus neoformans* was inoculated at 10^4^ and 10^6^ cells/mL in TCM as described above and incubated at 37°C in a 5% CO_2_ enriched atmosphere. After 18 h of incubation, the cultures were centrifuged and the supernatants collected to yield Titan-like Cell Supernatant (TCS) and regular cells supernatant (RCS). To evaluate the influence of these supernatants on the titan-like cell formation, *C*. *neoformans* cultures inoculated at 10^4^ cells/mL in 96 wells plates were prepared in different growth conditions: 1) Fresh TCM medium (TCM), 2) Supernatant from cultures of titan-like cells (TCS), 3) Supernatant from cultures of cells of regular size (RCS). These conditioned media were mixed with fresh TCM (1:1 proportion v/v). As control, we carried out a culture in which fresh TCM was diluted with the same volume of distilled sterile H_2_O. After the inoculation of the different media and mixtures with *C*. *neoformans* at 10^4^ and 10^6^ cells/mL and incubation at 37°C in a CO_2_ incubator for 18 h, the cell size was measured by microscopy as described above.

Chemical synthesis of the peptides was done by the proteomic facility of the National Centre for Biotechnology (CSIC, Spain) using an Multipep automatic synthesizer (Intavis, Köln, Germany) and Fmoc-Amino Acid Wang resins (Merck, Darmstadt, Germany). After release from the resin, the peptides were purified by reverse-phase chromatography in a semipreparative HPLC system (Jasco, Tokio, Japan) with a C18 Kromaphase column (Scharlab, Barcelona, Spain). The fractions were analyzed by mass spectrometry and lyophilized until their use. We synthesized peptides described in [32]: Qsp1 (NFGAPG**G**AYPW), an inactive version of this peptide (NFGAPG**A**AYPW) and a scrambled Qsp1 peptide (AWAGYFPGPNG). The peptides were dissolved in sterile PBS at 1 mM, and their effect on titan-like cell formation was tested at 30 μM and 15 μM in TCM. The samples were incubated for 18 hours at 37°C with 5% of CO_2_ in 96 wells plates. After the incubation period, the cells were observed by optical microscopy and the body cell sizes were measured.

### Nuclei analysis in titan-like cells

*Cryptococcus neoformans* cells from H99 strain were cultured overnight in TCM at 10^4^ and 10^6^ cells/mL at 37°C with CO_2_ without shaking. After the confirmation of titan-like cell formations by optical microscopy, cells were washed with dH_2_O and fixed with 70% ethanol at 24°C for one hour followed by an overnight incubation at 4°C. After fixing, the cells were washed twice with RNAse A buffer (0.2 M Tris, pH 7.5, 20 mM EDTA) and treated with 10 μg/mL of RNAse A for 4 hours at 37°C. After the incubation, cells were washed twice with PBS, suspended in PBS and incubated overnight at 4°C. Next day, the cells were centrifuged and suspended in a 200 ng/mL solution of 4',6-diamidino-2-phenylindole (DAPI) and incubated in the dark for 10 minutes at room temperature to stain the nucleus. Then, the cells were washed, suspended in PBS and the fluorescence intensity of the nucleus were analyzed by flow cytometry. Cells were examined for cell size by forward scatter parameter (FCS) and granularity by side scatter parameter (SSC) using the BD LSRFortessa X-20 cytometer (BD, Bioscience). Two populations of titan-like and regular cells were delimited and, in each population, the fluorescence intensity of the DAPI staining in 10,000 cells was measured. Data obtained were analyzed with the BD FACSDiva (BD, Bioscience) and FlowJo 10.4.2 (Tree Star Inc, Ashland, Oregon) softwares. The nuclei of the *C*. *neoformans* H99 titan-like and regular cells stained with DAPI were also observed by conventional fluorescence in a Leica DMI3000B microscope and confocal microscopy using a Leica SP5 confocal microscope.

### Influence of the protein kinase C (PKC) inhibitors and rapamycin on the formation of titan-like cells

The influence of the PKC pathway in the formation of the titan-like cells was evaluated by the addition of four different inhibitors: calphostin C, staurosporine and bisindolylmaleimide I, and genistein (all from Calbiochem) that inhibits tyrosine kinase as a control. For this purpose, 10 μM, 5 μM and 1 μM of calphostin C, bisindolylmaleimide I and Genistein and 0.01 μM and 0.001 μM of staurosporine. In all cases, the final concentration of DMSO was 0.1%, which did not inhibit titan-like cell formation.

### Phagocytosis of titan-like cells *in vitro* with RAW 264.7 macrophages

RAW 264.7 macrophages were maintained in DMEM medium supplemented with heat-inactivated 10% fetal bovine serum (FBS, Hyclone-Perbi), 10% NCTC medium and 1% non-essential amino acids (Sigma-Aldrich, Steinheim, Germany). The day before the experiment, the macrophage monolayer was separated from the plate by pipetting and the cells were centrifuged at 1,265 g. Macrophage suspensions were prepared at 2.5x10^5^ cells/mL. Two hundred μL per well were inoculated into 96 well plates and incubated overnight at 37° C and 5% CO_2_. The next day, different types of cells (titan-like cells obtained *in vitro*, and cells of regular size) at a final concentration of 5x10^5^ cells/mL with 5 μg/mL of monoclonal antibody 18B7 [76] were added to the macrophages for 2 hours. Phagocytosis was quantified by two different methods. First, the plates were observed with a Leica DMI 3000B microscope, and the percentage of infected macrophages was determined visually. In some cases, the plate was visualized with a Leica 4000B with a chamber that allowed incubation at 37°C and 5% CO_2_, and videos were taken as explained above. Alternatively, we quantified the phagocytosis percentage by flow cytometry. In this approach, we performed phagocytosis assays as above, but using larger volumes in 24-well plates containing 1 mL of medium. To differentiate yeast cells, we used a H99 strain that expresses the green fluorescence protein (H99-GFP) [77]. In some experiments, macrophages were exposed for 1 h to titan-like cells (H99 strain, 1:1 ratio) with mAb 18B7. As control, macrophages were also preincubated with the same cells, but without mAb, and also with medium alone (with and without mAb). Then, the wells were washed with fresh medium, and cryptococcal cells of regular size (H99-GFP, grown overnight in Sabouraud at 30°C) were added at 1:1 ratio for 2 h. After the incubation, we washed the plates, and separated the macrophages by continuous pipetting. The cell suspensions were washed and suspended in PBS containing 1% FCS. Macrophages were blocked with anti-Fc mAb (2.4G2, BD Biosciences, 5 μg/mL) for 10 min at 4°C. After that, the macrophages were washed with PBS/FCS, and then incubated with an anti-Mac1 mAb (anti-CD11b/Mac1-PE/Cy7, BioLegend, 1 μg/mL) for 20 min at 4°C in the dark. Finally, the cells were washed and suspended in 4% p-formaldehyde prepared in PBS. The cells were analyzed in a FACS Canto cytometer (Biosciences, California, EEUU) using FASCDiva software (versión 6.1). The phagocytosis percentage was calculated as followed: (number of PE-Cy7^+^/GFP^+^)/(total number of the PE/ PE-Cy7^+^ cells)*100. Experiments were performed in triplicate in three times on different days.

### Confocal microscopy

In some experiments, cryptococcal cells were labeled with mAb 18B7 labeled with Alexa-488 [78] at 1 μg/mL to label the capsule, and calcofluor (10 μg/mL, Sigma Aldrich) to label the cell wall. The cells were incubated for 1 h at 37, and then, they were washed and observed in a confocal SP5 Leica microscope.

### RNA preparation for RNASeq and transcriptomic data analysis

RNA extraction was performed using Trizol (TRI Reagent, Sigma Aldrich) with some modifications. After incubation of the cells, Trizol was added to the samples immediately and maintained in ice. Cells were broken during 5 minutes with FastPrep -24 (MP^TM^), alternating 20 seconds beating with 1 minute on ice. The RNAs concentration and quality was determined with a Nanodrop 8000 Spectrophotometer (Thermo scientific). RNA samples (0.1 μg/μL) were treated with DNase using the DNA-free kit (Thermo Fisher Scientific). Then, the RNA samples were purified using RNeasy Mini Kit (Qiagen).

Total RNA samples (0.5–1 μg) were treated to remove rRNA using Ribo-Zero Magnetic Kit (Epicentre, Illumina, San Diego, CA) according to the manufacturer's instructions. Then, mRNA was processed for library preparation using ScriptSeq v2 RNA-Seq Library Preparation kit (Epicentre, Illumina, San Diego, CA). Libraries were quantified using the QuantiFluor RNA System (Promega) and the quality and average size was determined using an Agilent 2100 Bioanalyzer. An Illumina NextSeq 500 High Output (400 M reads, 1x75 cycles) was using for sequencing. The FastQ files generated by the equipment were analyzed for low quality reads and those with Q<30 were removed. Original FastQ documents were uploaded to GEO repository (series record GSE111400). Mapping was carried out with the Bowtie2 software [79] using the *C*. *neoformans* var. grubii H99 genome as reference. The SAM files generated by Bowtie2 were analyzed using the software SeqMonk v0.39.0 (Babraham Bioinformatics). Three biological replicates for cells treated for 7 h (comprising 8.3 to 9.5E+06 reads each) and two for 18 h (6.9 and 7.9E+06 reads each) were combined, subjected to the RNA-Seq pipeline and evaluated for differential expression using the DESeq2 algorithm [80] with a FDR of p<0.05. The products of this test were recovered as *Annotated Probe Report*, which included the coding sequences (overlapping option), and processed in Excel format. To identify functional families significantly overrepresented among the differentially expressed genes, the lists of genes was submitted to Gene Ontology (GO) and FunCat analyses at the FungiFun server (https://elbe.hki-jena.de/fungifun/fungifun.php) [81]. To this end, unless otherwise stated, a Fisher's exact test with significance level set to 0.05 and the Benjamini-Hochberg adjustment method were used. Due to the large number of cryptococcal gene products lacking functional annotation in the databases, a Blastp search was conducted against the proteomes of several different, relatively well-annotated fungi, in order to expand the available functional information on the relevant genes.

### Ethics statement

All the animal procedures were approved by the Bioethical Committee and Animal Welfare of the Instituto de Salud Carlos III (CBA2014_PA51) and of the Comunidad de Madrid (PROEX 330/14) and followed the current Spanish legislation (Real Decreto 53/2013).

### Statistical analysis

Statistical analysis was performed with GraphPad Prism 5. Before comparison among groups, the normality of each simple was assessed using the Kolmogorov-Smirnov test (non-normal distribution when p<0.1). When normal distribution was assumed, differences were estimated using ANOVA and T-Student. For non-parametric distributions, the Kruskal-Wallis and Mann-Whitney tests were used. Statistical significant is highlighted with asterisks in the figures as follows: p>0.05, not significant (ns); p<0.05 and >0.01 (*); p<0.01 and p>0.001 (**); p<0.001 and p>0.0001 (***); p<0.0001 (****).

## Supporting information

S1 Fig To confirm nuclear analysis, we also used the *C*. *neoformans* strain SL305 Kn99α NOP1-mCherry [33] that express the nucleolar protein Nop1 tagged with mCherry. In this case, we prepared suspensions of *C*. *neoformans* cells at 10^4^ and 10^6^ cells/mL in 30 mL of TCM cell. After the incubation period, the cells were collected by centrifugation, fixed with 4% p-formaldehyde and analyzed by flow cytometry using the BD LSRFortessa X-20cytometer (BD, Bioscience). Two populations of titan-like and regular cells were delimited and, in each population, the fluorescence intensity of the NOP1-mCherry protein in 10,000 cells was measured. Data obtained were analyzed with the software BD FACSDiva (BD, Bioscience) and FlowJo 7.6.1 software (Tree Star Inc, Ashland, Oregon). Nuclei of the *C*. *neoformans* SL305 titan-like and regular cells were also observed by confocal microscopy using a Leica SP5 confocal microscope. In this case, cells were stained with mAb 18B7 conjugated to Alexa-488. A) Titan-like cells; B) cells of regular size. C. Histogram analysis of the NOP1-mCherry fluorescence intensity from cells of regular size (blue) or titan-like cells.
(TIF)

S2 Fig Effect of mixture of a and α strains on titan-like cell formation. Cells from different a and α pairs (A, 3259/3260; B, JEC20/JEC21; and C, NE822/NE824) were placed in TCM at 10^4^ cells/mL. In addition, parallel cultures in which the medium was inoculated with a mixture of same amount of cells (yielding a 10^4^ cells/mL concentration too) of both mating types were examined. The plates were incubated at 37°C with 5% CO_2_ without shaking for 18 h. Then, pictures were taken, and the size of around 50–100 cells was measured and plotted.
(TIF)

S3 Fig Cells grown in liquid Sabouraud or incubated in TCM at 10^6^ cells/mL or 10^4^ cells/mL (titan-like cells) were labeled with mAb 18B7 conjugated to Alexa-488 to visualize the capsule, and with calcofluor white to stain the cell wall (see Material and Methods). Then, the cells were observed in a confocal microscope, and brightfield and fluorescence images were taken. The bars denote 10 microns in all pictures. At the right panel, cells grown and labeled in the same way were analysed by flow cytometry to quantify the fluorescence intensity of the mAb. The corresponding dot plots and fluorescence histograms are shown. In the histograms, the fluorescence of control cells (without mAb) or with mAb Alexa-488 (18B7) are shown with arrows. The fluorescence image and cytometry analysis for each sample are aligned in the same rows.
(TIF)

S1 Video Cells in Sabouraud medium at 37°C. Videos were obtained as described in Material and Methods. Pictures were taken every 3 minutes, and video was assembled at 12 frames per second, so one second of video corresponds to 36 minutes of real time. Time in each frame reflects the incubation time in TCM.
(AVI)

S2 Video Development of titan-like cells. The yeasts were inoculated in TCM at and incubated overnight at 37°C with 5% CO_2_. Previous to the start of the acquisition, the cells had been in TCM for 8 h, so the time shown in each frame reflects the total incubation time since the cells were placed in TCM. Videos were assembled as described in S1 Video.
(AVI)

S3 Video Intracellular features in titan-like cells. Previous to the start of the video, the cells had been in TCM for 7h 30 min, so the time shown in each frame reflects the total incubation time since the cells were placed in TCM. Images were collected and processed as described above.
(AVI)

S4 Video Phagocytosis of titan-like cells by RAW264.7 macrophages. Videos of the interaction between titan-like cells and macrophages were performed as described in Material and Methods. Arrow highlights a titan-like cell. Time in the upper right part reflects the time since the beginning of the phagocytosis in minutes.
(AVI)

S5 Video Phagocytosis of cells of regular size obtained in TCM by RAW264.7 macrophages. Cryptococcal cells were incubated in TCM at a cell density of 10^6^ cells/mL, and after one night of incubation at 37°C with 5% CO_2_, phagocytosis was performed as described in material and methods. Arrow highlights phagocytosis events. Time in the upper right part reflects the time (min) since the beginning of the phagocytosis.
(AVI)

S6 Video Phagocytosis of cells of regular size obtained in Sabouraud by RAW264.7 macrophages. Cryptococcal cells were incubated in liquid Sabouraud and after one night of incubation at 37°C with 5% CO_2_, phagocytosis was performed as described in Material and Methods. Arrow highlights phagocytosis events. Time in the upper right part reflects the time since the beginning of the phagocytosis in minutes.
(AVI)

S1 Table List of genes induced during titan-like cell formation. Gene expression changes in titan-like cells isolated *in vitro*. Titan-like cells were obtained *in vitro* as described in Material and Methods, and transcriptomic analysis was performed by RNAseq. The table contains the genes that were significantly upregulated (using DESeq2 statistics) at 7 or 18 h of incubation in TCM. P value and fold change are also shown.
(XLSX)

S2 Table Repressed genes during titan-like cell formation. Same as S1 Table, but repressed genes (p<0.05) are listed.
(XLSX)
