# Supplementary figures and images for: Cryptococcus neoformans can form titan-like cells in vitro in response to multiple signals
Source: PLoS Pathog. 2018 May 18;14(5):e1007007. doi: 10.1371/journal.ppat.1007007 (PMC5959073; doi:10.1371/journal.ppat.1007007)

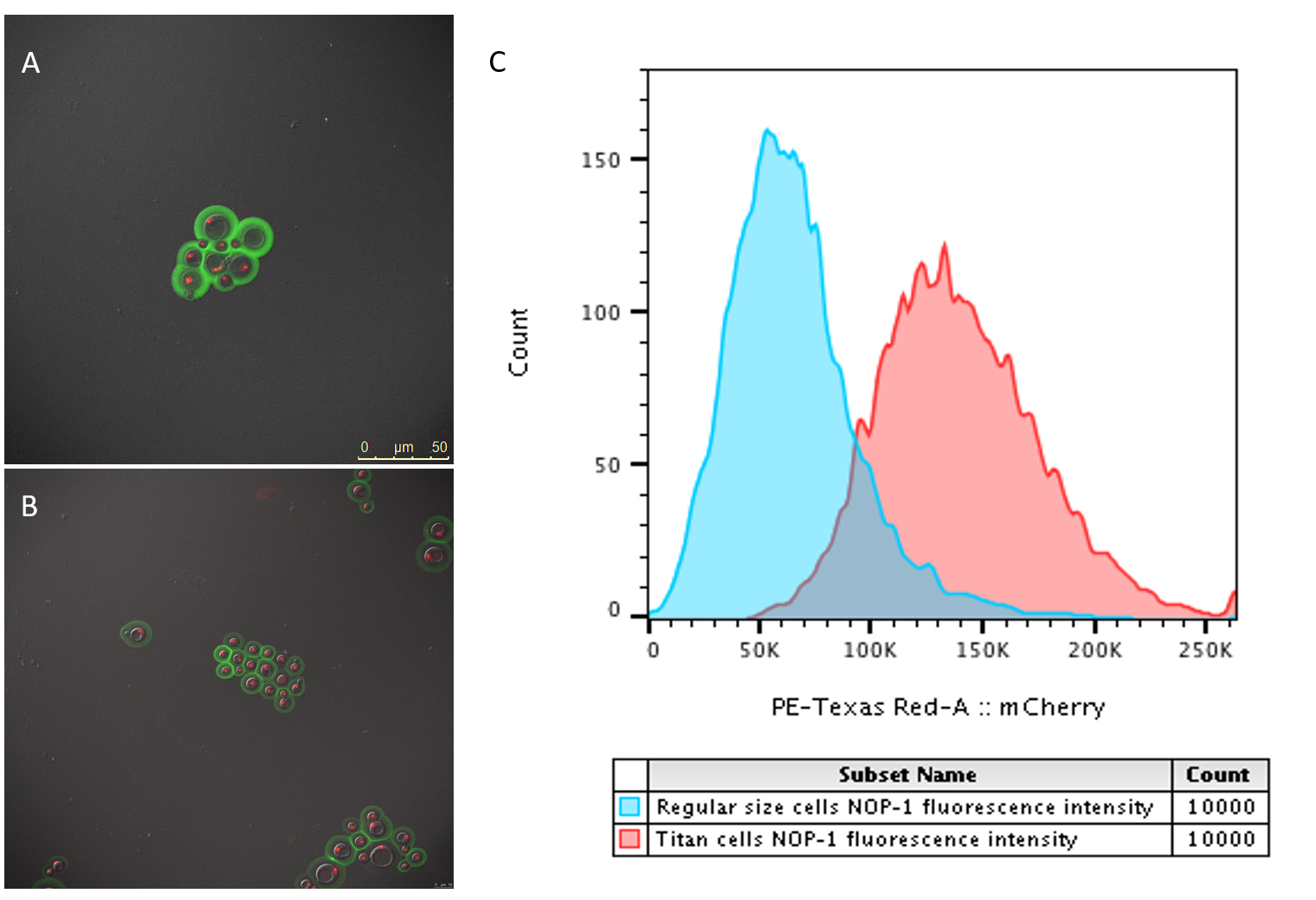

Supplement: S1 Fig — In this case, we prepared suspensions of C. neoformans cells at 104 and 106 cells/mL in 30 mL of TCM cell. After the incubation period, the cells were collected by centrifugation, fixed with 4% p-formaldehyde and analyzed by flow cytometry using the BD LSRFortessa X-20cytometer (BD, Bioscience). Two populations of titan-like and regular cells were delimited and, in each population, the fluorescence intensity of the NOP1-mCherry protein in 10,000 cells was measured. Data obtained were analyzed with the software BD FACSDiva (BD, Bioscience) and FlowJo 7.6.1 software (Tree Star Inc, Ashland, Oregon). Nuclei of the C. neoformans SL305 titan-like and regular cells were also observed by confocal microscopy using a Leica SP5 confocal microscope. In this case, cells were stained with mAb 18B7 conjugated to Alexa-488. A) Titan-like cells; B) cells of regular size. C. Histogram analysis of the NOP1-mCherry fluorescence intensity from cells of regular size (blue) or titan-like cells. (TIF) [file ppat.1007007.s001.tif]

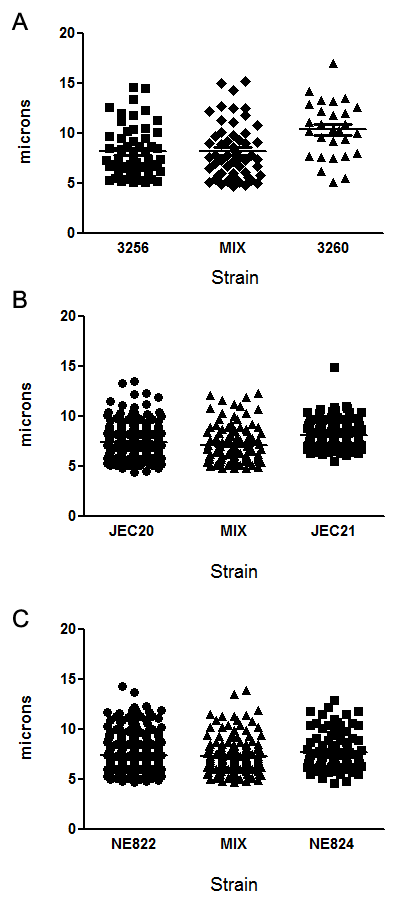

Supplement: S2 Fig — Cells from different a and α pairs (A, 3259/3260; B, JEC20/JEC21; and C, NE822/NE824) were placed in TCM at 104 cells/mL. In addition, parallel cultures in which the medium was inoculated with a mixture of same amount of cells (yielding a 104 cells/mL concentration too) of both mating types were examined. The plates were incubated at 37°C with 5% CO2 without shaking for 18 h. Then, pictures were taken, and the size of around 50–100 cells was measured and plotted. (TIF) [file ppat.1007007.s002.tif]

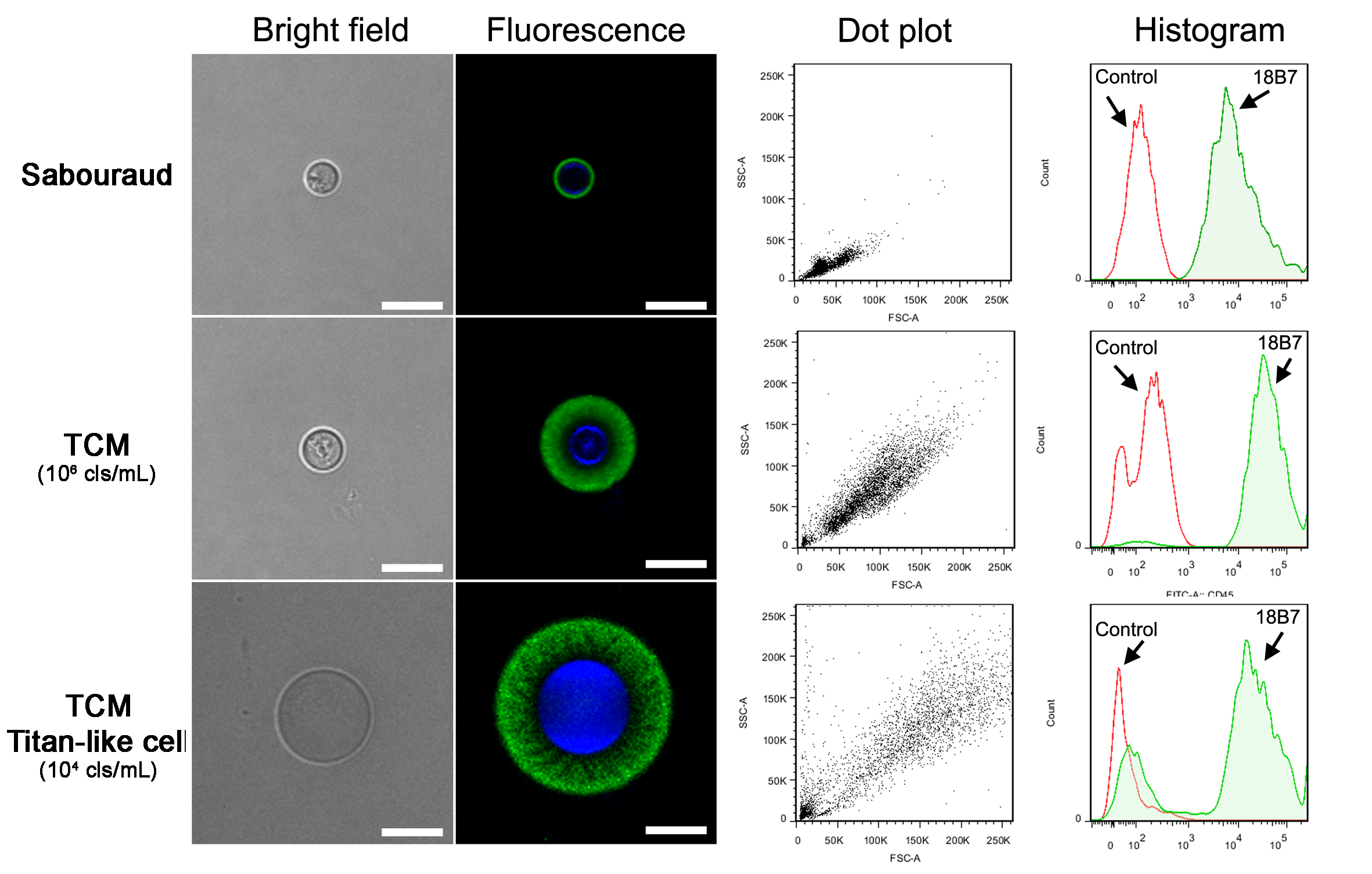

Supplement: S3 Fig — Then, the cells were observed in a confocal microscope, and brightfield and fluorescence images were taken. The bars denote 10 microns in all pictures. At the right panel, cells grown and labeled in the same way were analysed by flow cytometry to quantify the fluorescence intensity of the mAb. The corresponding dot plots and fluorescence histograms are shown. In the histograms, the fluorescence of control cells (without mAb) or with mAb Alexa-488 (18B7) are shown with arrows. The fluorescence image and cytometry analysis for each sample are aligned in the same rows. (TIF) [file ppat.1007007.s003.tif]
